# Endophyte-Promoted Phosphorus Solubilization in *Populus*

**DOI:** 10.3389/fpls.2020.567918

**Published:** 2020-10-21

**Authors:** Tamas Varga, Kim K. Hixson, Amir H. Ahkami, Andrew W. Sher, Morgan E. Barnes, Rosalie K. Chu, Anil K. Battu, Carrie D. Nicora, Tanya E. Winkler, Loren R. Reno, Sirine C. Fakra, Olga Antipova, Dilworth Y. Parkinson, Jackson R. Hall, Sharon L. Doty

**Affiliations:** ^1^Environmental Molecular Sciences Laboratory, Earth and Biological Sciences Directorate, Pacific Northwest National Laboratory, Richland, WA, United States; ^2^School of Environmental and Forest Sciences, College of the Environment, University of Washington, Seattle, WA, United States; ^3^Environmental Systems Graduate Group, University of California, Merced, Merced, CA, United States; ^4^Earth and Biological Sciences Directorate, Pacific Northwest National Laboratory, Richland, WA, United States; ^5^Advanced Light Source, Lawrence Berkeley National Laboratory, Berkeley, CA, United States; ^6^Advanced Photon Source, Argonne National Laboratory, Lemont, IL, United States

**Keywords:** *Populus*, poplar, endophytes, phosphorus, solubilization, synchrotron x-ray fluorescence, x-ray absorption near edge structure, x-ray computed tomography

## Abstract

Phosphorus is one of the essential nutrients for plant growth, but it may be relatively unavailable to plants because of its chemistry. In soil, the majority of phosphorus is present in the form of a phosphate, usually as metal complexes making it bound to minerals or organic matter. Therefore, inorganic phosphate solubilization is an important process of plant growth promotion by plant associated bacteria and fungi. Non-nodulating plant species have been shown to thrive in low-nutrient environments, in some instances by relying on plant associated microorganisms called endophytes. These microorganisms live within the plant and help supply nutrients for the plant. Despite their potential enormous environmental importance, there are a limited number of studies looking at the direct molecular impact of phosphate solubilizing endophytic bacteria on the host plant. In this work, we studied the impact of two endophyte strains of wild poplar (*Populus trichocarpa*) that solubilize phosphate. Using a combination of x-ray imaging, spectroscopy methods, and proteomics, we report direct evidence of endophyte-promoted phosphorus uptake in poplar. We found that the solubilized phosphate may react and become insoluble once inside plant tissue, suggesting that endophytes may aid in the re-release of phosphate. Using synchrotron x-ray fluorescence spectromicroscopy, we visualized the nutrient phosphorus inside poplar roots inoculated by the selected endophytes and found the phosphorus in both forms of organic and inorganic phosphates inside the root. Tomography-based root imaging revealed a markedly different root biomass and root architecture for poplar samples inoculated with the phosphate solubilizing bacteria strains. Proteomics characterization on poplar roots coupled with protein network analysis revealed novel proteins and metabolic pathways with possible involvement in endophyte enriched phosphorus uptake. These findings suggest an important role of endophytes for phosphorus acquisition and provide a deeper understanding of the critical symbiotic associations between poplar and the endophytic bacteria.

## Introduction

Crop productivity is constrained by the bioavailability of water-soluble nutrients, especially phosphorus (P) in the form of phosphate. The efficiency of P acquisition, in which fine roots play a critical role, is important in addressing global food and bioenergy security issues that arise from increasing world population and climate change. In nutrient-limiting environments, plants are known to form associations with microorganisms capable of increasing the bioavailability of nutrients (Chhabra and Dowling, 2017). Phosphorus complexes with calcium, iron, and aluminum in soils, causing the bio-availability of this essential macronutrient to be low. Phosphorus must be in the form of orthophosphate anions for plants to uptake (Chhabra et al., 2013). Mycorrhizal fungal symbiotic associations with plants have long been known to increase transport of P (Clark and Zeto, 2000). However, it is becoming clear that bacterial associations can also increase nutrient acquisition (Richardson, 2001; Compant et al., 2010; Gaiero et al., 2013; Vandenkoornhuyse et al., 2015; Chhabra and Dowling, 2017). A wide variety of non-nodulating plant species are able to thrive in low-nutrient settings through symbiosis with internal microorganisms called endophytes (Santi et al., 2013). There has been a body of research on endophytes suggesting their important role in the growth of plants under nutrient-limiting conditions (Hardoim et al., 2008; Ryan et al., 2008; Gaiero et al., 2013).

Microbial mechanisms for solubilization of P include production of organic acid anions such as gluconic acid (Crespo et al., 2011; Oteino et al., 2015), malic acid (Jog et al., 2014), citric acid (Chen et al., 2014; Hane et al., 2014), salicylic acid, and benzeneacetic acid (Chen et al., 2014), as well as oxalic acid (Schneider et al., 2010; Mendes et al., 2014). Pyrroloquinoline quinone (PQQ) is involved in solubilization of both organic and inorganic phosphates as it is a redox cofactor of glucose dehydrogenase (GDH) for oxidation of glucose to gluconic acid (Misra et al., 2012). While screens for potentially symbiotic traits of plant-associated bacteria often include testing for solubilization of tricalcium phosphate [Ca_3_(PO_4_)_2_], it is valuable to also test for the rarer ability to solubilize Fe-phosphate and Al-phosphate (Bashan et al., 2013).

Poplar (*Populus*) trees are early successional tree species that are known to be able to grow in nutrient-limited environments (Doty et al., 2016). Poplar, a member of the Salicaceae family, is considered important as a wood product, for bioenergy and for environmental services (Bradshaw and Stettler, 1995; Isebrands and Richardson, 2014). While it has been demonstrated that P-solubilizing bacteria can provide P that impacts plant growth (Chhabra et al., 2013; Oteino et al., 2015), such studies have not been done on ecologically important poplar trees. Recently, the role of endophytic bacteria in nitrogen (N) fixation was reported in wild poplar (Doty et al., 2016). Although many of the endophyte strains are shown to solubilize Ca_3_(PO_4_)_2_ (Kandel et al., 2017), how plants interact with phosphate solubilizing endophytic bacteria remains understudied.

In this study, we tested the hypothesis that, apart from mycorrhizal networks, closely associated endophytic bacteria also contribute significant P to the host plants. We report evidence of endophytic activity to promote P uptake by comparing samples inoculated with two P-solubilizing endophytes and those uninoculated (controls). By comparing root masses from physical root measurements as well as root imaging by x-ray microtomography, we show indications of increased nutrient uptake in the plants inoculated with the endophytes. P inside the roots was visualized using synchrotron x-ray fluorescence (SR-XRF) microscopy. Using P K-edge x-ray absorption near edge structure (XANES), we determined P speciation inside the roots. Finally, our proteomics analysis points to differential protein enrichment between inoculated and uninoculated poplar roots as well as proteins that may have been involved in P solubilization and utilization. These findings suggest that endophytes are important for P acquisition and give us a deeper understanding of the biological relevance of the symbiosis between the plants and the endophytic bacteria.

## Materials and Methods

### Microbial Strains

Diazotrophic endophyte strains from poplar (Doty et al., 2009; Khan et al., 2015; Kandel et al., 2017) had previously been screened for Ca_3_(PO_4_)_2_ solubilization on National Botanical Research Institute’s phosphate (NBRIP) growth medium plates as described before (Nautiyal, 1999; Khan et al., 2015). Strains with the highest phosphate solubilization index were further screened for the ability to grow on medium with aluminum phosphate or iron phosphate as the sole P sources. Based on these screens, strains WP5 (*Rahnella* sp.; Khan et al., 2015) and WP42 (*Burkholderia* sp.; Kandel et al., 2017) were selected for further study. All bacteria, and controls, for this experiment were incubated at 30°C with shaking at 150 rpm.

### Quantification of Bacterial Growth and Phosphate Solubilization

Modified phosphate-free NBRIP liquid medium was prepared, with glucose 20 g/L and magnesium chloride 10 g/L as described for maximum phosphate solubilization (Nautiyal, 1999), and termed enhanced NBRIP (ENBRIP) broth. ENBRIP contained per liter: 20 g glucose, 5 g Ca_3_(PO_4_)_2_, 10 g magnesium chloride hexahydrate (MgCl_2_·6H_2_O), 0.25 g magnesium sulfate heptahydrate (MgSO_4_·7H_2_O), 0.2 g potassium chloride (KCl), and 0.1 g ammonium sulfate [(NH_4_)_2_SO_4_]. Fifty milligram of either Ca_3_(PO_4_)_2_, iron phosphate, or aluminum phosphate was added to 125-ml Erlenmeyer flasks containing 10 ml of ENBRIP broth prior to autoclaving at 121°C for 25 min. The pH of the medium was adjusted to 7.0 prior to autoclaving. Starting cultures of strains WP5 and WP42 were grown overnight in 25 ml ENBRIP containing 3 g/L potassium phosphate as well as in 25 ml MG/L broth (Cangelosi et al., 1991) to ensure sufficient cells for the assays. The MG/L broth contained per liter: 5 g tryptone, 1.5 g yeast extract, 5 g sodium chloride (NaCl), 10 g mannitol, 2.32 g sodium glutamate, 0.5 g monobasic potassium phosphate (KH_2_PO_4_), and 0.2 g MgSO_4_·7H_2_O. Cells were washed three times in ENBRIP without phosphate by centrifugation at 6,000 rpm for 20 min at 15°C. The cells were then grown for several hours in ENBRIP to allow depletion of residual internal phosphate. The optical density at 600 nm of the washed cells were then measured by spectroscopy (Fisher) and the amount of culture needed to give an optical density of 0.1 in 10 ml was calculated. The flasks of ENBRIP with added phosphates, and a set without added phosphate, were inoculated in triplicate with each strain. Another set of 12 uninoculated control flasks was prepared, without the addition of cells. All flasks were incubated for 3 days before the cultures were transferred into 50-ml conicle tubes and allowed to settle for 90 min. Solubilized phosphate was quantified using the method of Murphy and Riley (1962) modified as follows: 5 μl of the settled cultures was added to 795 μl water and reacted with 200 μl of freshly prepared reagent B. Absorbance was read at 880 nm. Optical density of the settled cultures was read at 600 nm as an estimate of cell density.

### Localization of the Endophyte Strains on Poplar Roots

Fluorescent tags were introduced into strains WP5 and WP42 through electroporation using standard protocols (Cangelosi et al., 1991). The green fluorescent protein (GFP) plasmid, pBHR-GFP (Stevens et al., 2005), conferring kanamycin resistance, and the purple fluorescent protein, pMMB67EH/Kate2, conferring gentamicin resistance, were obtained from the Greenberg and Miller labs (UW Microbiology Department). Tagged strains were verified by fluorescent microscopy using a Zeiss Imager M2 equipped with an AxioCam MRM and recorded with Zeiss Zen software (Karl Zeiss, LLC, Thornwood, NY, United States). Rooted, internally-sterile *Populus trichocarpa* clone Nisqually-1 poplar plants were co-cultivated in sterile 40 ml vials for 2 days with the tagged strains that had been brought to an OD_600_ of 0.5 in half-strength Murashige and Skoog (MS) medium without sucrose (Murashige and Skoog, 1962). Plants were then washed three times in sterile water and placed into fresh 0.5X MS. Colonization of root surfaces was visualized by fluorescent microscopy.

### Plant Growth and Conditioning for P Solubilization Studies

Internally sterile *P. trichocarpa* clone Nisqually-1 plants were propagated on McCown’s Woody Plant Medium (Phytotechnology Labs). Twelve-day old apical cuttings with fresh roots were transferred to Magenta vessels (Caisson Labs) containing 10 ml of ¼ strength MS broth without sucrose (pH 5.8). Endophyte strains, WP5 and WP42, were grown in N-Limited Combined Carbon Medium (Rennie, 1981) for 2 days. Colonies were inoculated into 10 ml NL-CCM broth in 125-ml flasks and grown on a shaker at 30°C for 2 days. Cells were pelleted by centrifugation and resuspended in N-free medium (Doty et al., 2009). Inoculum was prepared with a mixture of the two strains at equal densities to a final OD600 of 0.1 in ¼ X Hoagland’s solution (Hoagland and Arnon, 1938). Half of the plants were co-cultivated in the inoculum while the control plants received the sterile broth. As soil medium, dried sand was thoroughly mixed with Ca_3_(PO_4_)_2_ (Sigma-Aldrich) in a 200:1 ratio by weight before use (Oteino et al., 2015). The non-water soluble Ca_3_(PO_4_)_2_ was chosen to confirm the capability of the endophytes to help the plant mobilize that compound. The ¼ X Hoagland’s Solution (Hoagland and Arnon, 1938) was added to the soil, and the poplar plants were transferred into it. The samples that contained the endophyte strain were called P-mix samples, and those that did not were called Control samples. Plants were grown for 4 weeks in Magenta GA-7 vessels in growth chambers under 16 h/8 h light-dark regime (with light intensity of 250 μmol m^−2^ s^−1^), temperature of 24°C day/18°C night, and relative humidity of 60%. A subset of plants grown under same condition were used for x-ray computed tomography (XCT) and μXRF/μXANES analyses (see below).

### Soil Analysis

The same sandy soil mix was used for planting all P-mix and Control samples. In order to evaluate whether there was a significant difference between the total P concentrations of the P-mix and Control samples after the plants were harvested, Inductively Coupled Mass Spectrometry (ICP-MS) measurements were carried out on one soil sample from the P-mix group and one from the Control group. For acid extraction to bring all of the P into solution for the analysis, 20 g of sediment was weighed into 50-ml centrifuge tubes. Fifteen microliter of 2% nitric acid (trace metal grade) was added to the centrifuge tubes and vortexed for 24 h. The samples were then put in the 4°C fridge to let the solids settle. The supernatant was pulled off the next morning and all samples were left at 4°C until analysis.

### Root Collection Procedure

Ten plants (five Control and five P-mix) were collected for root biomass, morphology analysis, and further proteomics characterization. The clippers and forceps were wiped down with ethanol between collecting each sample. Plants were gently removed from the planting boxes by tilting the boxes and rocking back and forth to expose roots. While holding onto the plant, the roots were gently freed from sand and gently rinsed with deionized water to remove the remaining sand. The plants were placed on large Kim-wipes to remove excess moisture and the longest root length was measured. The plant leaf mass was subsequently weighed and recorded. A picture of the plant was taken, then the roots were cut off, weighed, put in a labeled 50-ml Falcon tube and flash-frozen in liquid nitrogen. The roots were then placed in a −80°C freezer for proteomics analysis.

### Proteomics Characterization

#### Protein Extraction and Digestion

Roots were flash frozen in liquid nitrogen and ground into a fine frozen powder using a Freezer/Mill (SPEX, Metuchen, NJ, United States) using a program that ran 2 cycles, with a 1 min precool, and a 2 min run time at 10 cycles/min. Two milliliters of ice-cold methanol was added to about 0.5–1 ml volume of frozen plant powder, and the sample was vortexed. Then, 1 ml of nanopure water and 1.8 ml of chloroform were added and the sample was shaken vigorously for about 15 s into an emulsion. Each sample was centrifuged for 10 min at 4°C at 7,197 × *g*. The middle protein pellet was washed with 3 ml ice cold methanol and centrifuged at 10,000 × *g* for 10 min at 4°C three times to remove remaining metabolites from protein sample. Excess methanol was removed by drying the pellets gently under a flow of nitrogen for ~2 min. A protein solubilization solution containing 7 M urea, 2 M thiourea, 4% CHAPS, and 5 mM TCEP was added to completely cover each pellet, plus 500 μl more. Samples were then incubated at 4°C overnight. Debris/protein pellets from each sample were physically mixed into solution with a pipette tip and the slurry sonicated briefly in a sonoreactor. The protein slurries were then incubated at 60°C for 30 min, with samples vortexed and sonicated in a sonoreactor again for about 30 s. Each sample was then centrifuged for 10 min at 5,000 × *g* at 4°C. A Coomassie Plus protein assay (Pierce, Rockford, IL, United States) using a bovine serum albumin standard (BSA) was next performed on the individual supernatants to estimate protein concentration. Afterwards, the denatured samples were diluted tenfold with 50 mM ammonium bicarbonate, pH 8.0. CaCl_2_ was added to a concentration of 2 mM and trypsin (Affymetrix, Santa Clara, CA, United States) was added at a trypsin:sample ratio of 1:50 (w/w). Samples were digested overnight at 37°C and alkylated with chloroacetamide at a concentration of 5 mM in the dark at 37°C for 30 min. The peptides from each treatment were desalted with C-18 SPE columns (SUPELCO Discovery) using a 0.1% TFA in nanopure water to wash the peptides and 80:20 acetonitrile:water with 0.1% TFA solvent was used to elute the peptides. Peptides were then quantified using a BCA assay (Pierce, Rockford, IL, United States) with a BSA standard.

#### iTRAQ Peptide Labeling

Peptides were labeled with 10-plex tandem mass tag (TMT) reagents (ThermoScientific, San Jose, CA, United States) according to manufacturer’s instructions. After samples were labeled, they were dried down in a centrifugal vacuum concentrator.

#### Offline Fractionation of Peptides and Preparation of Proteome Samples

Labeled peptides were separated using an off-line high pH (pH 10) reversed-phase (RP) separation with a Waters XBridge C18 column (250 mm × 4.6 mm column containing 5 μm particles and a 4.6 mm × 20 mm guard column) using an Agilent 1200 HPLC System. The sample loaded onto the C18 column was washed for 15 min with Solvent A (10 mM ammonium formate, adjusted to pH 10 with ammonium hydroxide). The LC gradient started with a linear increase of Solvent B (10 mM ammonium formate, pH 10, 90:10 acetonitrile:water) to: 5% Solvent B over 10 min, 45% Solvent B over 65 min, and then a linear increase to 100% Solvent B over 15 min. Solvent B was held at 100% for 10 min, and then was changed to 100% Solvent A, this being held for 20 min to recondition the column. The flow rate was 0.5 ml/min. A total of 96 fractions were collected into a 96-well plate. The high pH RP fractions were then combined into 24 fractions using the concatenation strategy previously reported (Wang et al., 2011) excluding CHAPS containing wells (F2–F11). Peptide fractions were dried down and re-suspended in nanopure water at a concentration of 0.075 μg/μl for mass spectrometry analysis using an Q Exactive Hybrid Quadrupole Orbitrap Mass Spectrometer (Thermo Scientific) system as described below.

#### Mass Spectrometry

All peptide samples were analyzed using an automated constant flow nano LC system (Agilent) coupled to Q Exactive Orbitrap (Thermo Fisher Scientific). Electrospray emitters were custom made using 150 μm o.d. × 20 μm o.d. × 20 μm i.d. chemically etched fused silica. An on-line 4-cm × 360 μm o.d. × 150 μm i.d. fused-silica capillary analytical column (3 μm Jupiter C18) was used. Mobile phases consisted of 0.1% formic acid in water (A) and 0.1% formic acid acetonitrile (B) operated at 300 nl/min with a gradient profile as follows (min: %B); 0:5, 2:8, 20:12, 75:35, 97:60, and 100:85.

The LTQ Orbitrap Velos mass spectrometer was operated in the data-dependent mode acquiring higher-energy collisional dissociation (HCD) scans (*R* = 7,500, 5 × 104 target ions) after each full MS scan (*R* = 30,000, 3 × 106 target ions) for the top 10 most abundant ions within the mass range of 300–1,800 m/z. An isolation window of 2.5 Th was used to isolate ions prior to HCD. All HCD scans used normalized collision energy of 45 and maximum injection time of 1,000 ms. The dynamic exclusion time was set to 60 s and charge state screening was enabled to reject unassigned and singly charged ions.

#### Peptide Identification and Quantification

For peptide identification, MS/MS spectra were searched against a decoy *P. trichocarpa* v3.1 protein database from Phytozome^1^ using the algorithm SEQUEST (Eng et al., 1994). Search parameters included: no enzyme specificity for proteome data and trypsin enzyme specificity with a maximum of two missed cleaves, ±50 ppm precursor mass tolerance, ±0.05 Da product mass tolerance, and carbamidomethylation of cysteines and TMT labeling of lysines and peptide N-termini as fixed modifications. Allowed variable modifications were oxidation of methionine and proline. MSGF+ spectra probability values (Kim et al., 2008) were also calculated for peptides identified from SEQUEST searches. Measured mass accuracy and MSGF spectra probability were used to filter identified peptides to <0.4% false discovery rate (FDR) at spectrum level and <1% FDR at the peptide level using the decoy approach. TMT reporter ions were extracted using the MASIC software (Monroe et al., 2008) with a 10 ppm mass tolerance for each expected TMT reporter ion as determined from each MS/MS spectrum.

#### Protein Abundance Value and Significance Determination

Relative abundances of peptides were determined using TMT reporter ion intensity ratios from each MS/MS spectrum. Individual peptide intensity values were determined by dividing the base peak intensity by the relative ratio associated with each reporter ion. All peptide data were transformed to a log2 value, mean centered normalized, then each value was taken as an exponent of 2 to convert back to an unlogged value. Peptide abundance values were separated into two datasets, one of peptides unique to a single protein and peptides which may have been derived from two or more proteins. Peptides were rolled up to a protein value by summing the peptides that belong to each protein in each dataset. For the peptide data which could come from multiple proteins, these were concatenated into a group name to represent all proteins which could produce each unique peptide. Protein rollup calculations from the unique or shared/group peptide table were designated in the final protein rollup table. Kyoto Encyclopedia of Genes and Genomes (KEGG) protein function orthologs^2^ (Kanehisa et al., 2017), gene ontologies^3^ (Carbon et al., 2017), and other annotations were obtained from the *P. trichocarpa* v3.1 annotation file located at Phytozome^4^ (Goodstein et al., 2012). A partial least-squares and Pearson’s pairwise correlation plots were constructed using the proteomics software Inferno.^5^ Log2 values were uploaded to MeV^6^ (Wang et al., 2017), where a *t*-test was performed between Control and P-mix sample groups using a Welch approximation (assuming unequal group variances) and with a significance determined with a cut-off value of *p* less than 0.05 with a value of *p* based on a *t*-distribution.

#### STRING Network Analysis

A Search Tool for the Retrieval of Interacting Genes/Proteins (STRING): functional protein association network^7^ (Szklarczyk et al., 2019) was performed on the proteins determined to be significantly changed from the *t*-test (*p* < 0.05) against co-expression, co-occurrence, databases, gene fusion, experiments, neighborhood, and textmining active interaction sources found in the STRING databases for *P. trichocarpa*. The minimum required interaction score was 0.400, and disconnected nodes in the network were hidden from the network image.

### X-Ray Computed Tomography

To characterize root growth and architecture, inoculated and control samples were scanned using a microfocus XCT scanner (X-Tek/Metris XTH 320/225 kV, Nikon Metrology, Brighton, MI, United States). Scans were performed at 90 kV and 350 μA x-ray power. During scans, samples were rotated continuously with momentary stopping to collect each projection. A total of 2,000 projections were collected over 360° with an exposure time of 500 ms per projection. Images were collected at an isotropic voxel resolution of 40.0 μm, resulting in 32-bit gray-scale images. The raw images were reconstructed to create a three-dimensional (3D) dataset using the software CT Pro 3D (Nikon Metrology, Brighton, MI, United States). For higher resolution imaging, XCT data were collected at beamline 8.3.2 of the Advanced Light Source (ALS) at Lawrence Berkeley National Laboratory (LBNL). A double-multilayer monochromator was used to select 17 keV x-rays, and detection used a 0.5 mm LuAG scintillator and 2× lenses with a sCMOS PCO.Edge camera, giving a 3.3 μm pixel dimension, and a 8.4 mm horizontal field of view. The sample to scintillator distance was 15 mm. A 300 ms exposure time yielded 8,000 counts on the 16-bit camera (allowing a maximum of 65,535 counts). For each tomographic scan, 1,313 projections were acquired over a 180° rotation, with a total scan time of 15 min. The images were analyzed using Avizo (Thermo Fisher Scientific, Waltham, MA, United States) to segments the roots and generate root volume and surface area data.

### Synchrotron X-Ray Fluorescence Microprobe

Petrographic thin sections (30-μm thick) of root samples were prepared by Spectrum Petrographics, Inc. Soda-lime petrographic glass slides of size 27 × 46 × 1.2 mm were mounted with a UV curing acrylic adhesive. High-purity fused quartz glass slides with superglue mounting adhesive were used for synchrotron sample preparations. The slides ranged in thickness from 0.030 to 0.040″. High-purity fused quartz cover glasses of about 200 μm thick were used to mount the samples with permanent adhesive. That sample/coverglass was then mounted in “piggyback” style with superglue (removable with acetone) onto a normal petrographic slide that had the necessary rigidity for further processing. Another set of samples were mounted on Si_3_N_4_ windows (5 × 5 mm size, 2 μm thick, Norcada Inc., Edmonton, Canada) after the following preparation. Roots from P-mix and Control samples were gently washed with MilliQ water. Root tips were then harvested and frozen at −80°C in 2.5% CMC using a 600-μl Eppendorf tube. Samples were then kept in the −80°C overnight tip orientation down. Samples were removed from the tube and mounted onto a chuck with 200-μl of Milliq water for fixing. Samples were cryosectioned on a Thermo NX-70 with the chuck set to −14°C and the blade set to −11°C. 60-μM sections were sliced both longitudinally and laterally to the tip of the root. Using tweezers kept at −20°C, sections were then placed onto the Si_3_N_4_ windows and thaw-mounted.

X-ray microprobe analyses were carried out at the ALS XFM beamline 10.3.2 at LBNL (Marcus et al., 2004). All data were recorded in fluorescence mode at room temperature, using a Si(111) monochromator, an Amptek FAST SDD fluorescence detector, and a He-filled chamber in the sample-detector path. Root and P reference powder samples were mounted on Magic Scotch 3 M tape, found to have the lowest detectable P content (no P, Si, or S detected). μXRF mapping was performed at an incident energy of 100 eV above the P K-edge (2,245 eV). This allowed for capturing the distribution of P as well as Ca and K (through harmonics), important elements in soils and roots. Coarse μXRF maps of the roots were first recorded with 35 μm pixel size, then specific regions of interest mapped at higher resolution (8 μm). XRF maps were displayed to the same intensity scale and analyzed for P distribution and concentration. Defocused and microfocused P K-edge XANES data were collected along with the μXRF images on the same samples to investigate P speciation. A variety of inorganic P reference compounds were used: AlPO_4_, FePO_4_, and multiple Ca-bound phosphates (see Supplementary Figure S6). Organically bound P was represented by phytate, DNA, and lecithin. Reference compounds were acquired by this project and from other studies (Barnes et al., 2019; O’Day et al., 2020). Bulk and microfocus spectra were deadtime corrected and deglitched using a custom LabVIEW software available at beamline 10.3.2. Spectra were then imported to the ATHENA software package (Demeter 0.9.20; Ravel and Newville, 2005) for calibration, baseline correction, normalization and linear combination fitting (LCF). Spectra were corrected for over-absorption prior to LCF using a custom LabVIEW software available at beamline 10.3.2. Calcium phosphate (CaHPO_4_) with a white line at 2152.26 eV was used for calibration. Baseline correction and edge-step normalization parameters were varied for individual samples to reduce error associated with LCF (Werner and Prietzel, 2015). Fits were implemented with the component sum not forced to unity and a maximum of four reference compounds were allowed. A final fit was chosen based on the combination of reference compounds with lowest R-factor that also visually aligned with the unknown sample, and only fits within ±2.5% of 100% were accepted. Error associated with this technique is about 5–10% (Ajiboye et al., 2007; Werner and Prietzel, 2015), therefore, in instances where a reference fit with <5% of that compound, it was removed and the sample refit.

Additional high-resolution x-ray fluorescence data were collected at 2-ID-E beamline of the Advanced Photon Source (APS) at Argonne National Laboratory. X-ray energy was set to 10 KeV using 3.3 cm periodicity undulator and Si (111) crystal monochromator; beam was focused with Fresnel zone plate down to 400 × 400 μm spot on sample. Sample was placed in Helium and raster scanned using translation stages with 200 nm step in X and 300 nm step in Y and 10 msec/pixel at 45° angle to incident beam to minimize sample-to-detector distance and improve signal for low Z elements (P-Ca). Emitted x-ray spectra were recorded by four-element silicon-drift Vortex-ME detector (Hitachi), calibrated, and fitted by MAPS (Vogt, 2003) using thin-film standard AXO 1 X (AXO Dresden GmbH) to obtain 2D maps of elements.

The P concentrations inside the roots were determined from all high-resolution μXRF maps collected at beamline 2-ID-E at the APS. Using the average values, a final P concentration was generated to demonstrate any change in P content between P-mix and Control samples. The data were analyzed, fitted, and quantified using MAPS software (Vogt, 2003). The P concentrations were determined from two regions of interest (ROIs), from inside the root and substrate background from outside the root. In order to avoid soil traces within samples, ROIs were selected in areas with minimal interference from element, which are present in soil but have negligible uptake by plants, such as Si and Ti (van der Ent et al., 2018). In addition, other metals like Ca, K, and Fe, Mn, Cu, and Zn were measured from both the root and the background to correlate those with the P concentrations to see how it correlates with Ca, especially. The data points from each detector element for each region were normalized using downstream ion chamber and averaged. The substrate background elemental values were subtracted to get the true P concentration from inside the roots, as well as K, Ca, Fe, Cu, and Zn. Using the average values, a final P concentration was generated to demonstrate any change in P content between P-mix and Control samples.

## Results

### Phosphate Solubilization by the Endophyte Strains

**Figure 1:**
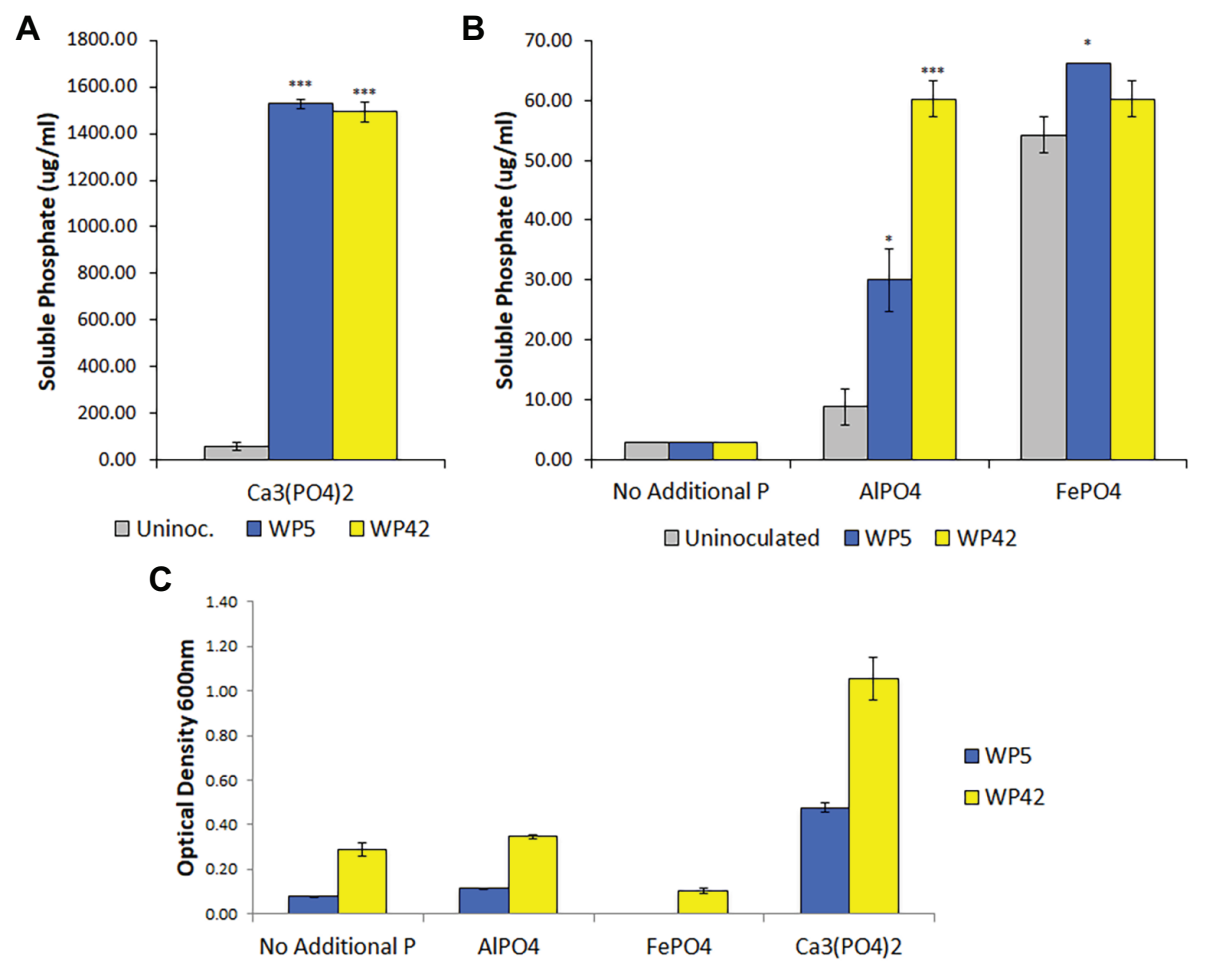
**(A,B)** Solubilized phosphate in enhanced National Botanical Research Institute’s phosphate (ENBRIP) media after 36 h incubation (*n* = 3, ±SE). Significantly different from uninoculated media (*t*-test, ^*^p < 0.05, ^***^p < 0.001). **(C)** Cell concentrations in ENBRIP media after 36 h incubation (*n* = 3, ±SE). Optical densities of uninoculated media were subtracted for each phosphate type to control for unsettled phosphate particulates.

Poplar endophyte strains, WP5 and WP42, were previously tested using plate assays for the ability to solubilize Ca_3_(PO_4_)_2_ (Khan et al., 2015; Kandel et al., 2017) and to grow on aluminum phosphate and iron phosphate (unpublished). To quantify bacterial growth and phosphate solubilization, liquid assays were performed. Both strains grew well only in the medium containing Ca_3_(PO_4_)_2_, but appeared to have negligible growth beyond controls in media containing aluminum phosphate (Figure 1C). Results of the colorimetric method to quantify phosphate indicated that both strains solubilized Ca_3_(PO_4_)_2_, with residual suspended solid phosphate seen in the control (Figure 1A). In addition, strains WP5 and WP42 both showed a limited ability to solubilize aluminum phosphate, with WP5 alone showing minor solubilization of iron phosphate above controls (Figure 1B). However, the cell concentration observed by optical density for both strains in iron phosphate was lower than the controls without added phosphate (Figure 1C).

### Root Colonization by the Endophyte Strains

**Figure 2:**
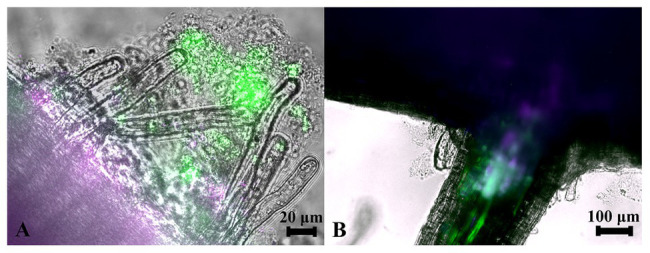
Colonization of poplar roots by WP42 [tagged with green fluorescent protein (GFP)] and WP5 [purple fluorescent protein (PFP)]. **(A)** Microbes colonizing the root surface, as a likely biofilm, covering the primary root (seen bottom left of image) as well as the root hairs which eminate from it. Microbial populations are, therefore, spatially available to manipulate external soil chemistry. Scale bar 20 μm. **(B)** The junction of a primary root (seen top of image) with a lateral root. By focusing beyond the epidermis, endophytic fluorescence can be observed inside root tissue showing special availability for the manipulation of internal chemistry as well. Scale bar 100 μm.

Since strains WP5 and WP42 were originally isolated as endophytes of poplar branches, we tested if they could also colonize root surfaces where their ability to solubilize phosphate from soils could be most advantageous to the host plant. Fluorescent microscopy images of poplar plants co-cultivated with WP5 tagged with purple fluorescent protein (PFP) and WP42 with GFP are shown in Figure 2, where colonization by the two strains is visualized simultaneously. Microbes, as a likely biofilm, are observed colonizing the root surface, covering the primary root as well as the root hairs. Microbial populations are, therefore, spatially available to manipulate external soil chemistry. When the junction of a primary root with a lateral root is investigated by focusing beyond the epidermis, endophytic fluorescence can be observed inside root tissue, showing availability for the manipulation of internal chemistry as well.

### Soil Phosphorus Content

The starting total P concentration in the sand that was used as the root inhabiting medium was calculated to be approximately 3,500 μg/kg, based on the nutrient mix added and the weight and volume of the sand used in the pots. At the time of root harvesting, from ICP-MS, the total P concentration for the sand that contained a P-mix sample was reduced to 1,690 μg/kg, while for the Control sample it was reduced only to 2,680 μg/kg (see Supplementary Figure S1). Results from this complementary analysis were consistent with increased microbial P mineralization uptake of soluble P by endophyte inoculated plants.

### Root Biomass and Morphology

We monitored poplar root phenotypic changes in P-mix inoculated and control plants. Roots and leaves from five biological replicates inoculated with the endophyte (P mix) and five Control samples were harvested for root length, mass, and leaf mass measurements prior to proteomics characterization. Representative Control and P-mix and the harvested plants are shown in Supplementary Figure S2. Root dry weight and root length showed similar values under both conditions and no significant changes were observed (see Supplementary Table S1).

### Root Imaging by XCT

**Figure 3:**
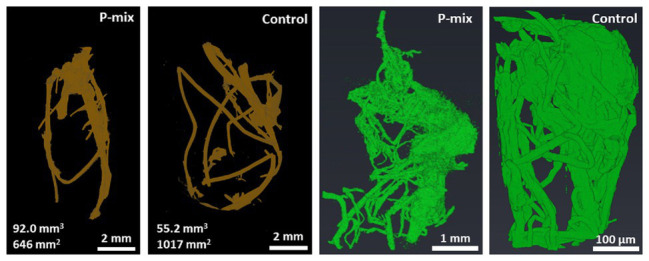
CT analysis of a whole root (root of brown color) showing a P-mix sample with 92.0 mm^3^ volume and 646 mm^2^ surface area, and a control sample with 55.2 mm^3^ volume and 1,017 mm^2^ surface area. Analysis of synchrotron micro-CT images (green color) shows increased fine root formation in the Control; P-mix sample with a root volume 538.3 mm^3^ exhibited a surface area of 1842.1 mm^2^, while a Control sample with 27.4 mm^3^ volume had a surface area of 2940.6 mm^2^.

Since the comparison of root dry weight and root length values of the P-mix and Control samples was inconclusive, we turned to 3D imaging to look at the architecture and microstructure of the roots in more details. Root volume and surface area measurements were carried out by tomographic image-based analysis. Whole roots imaged by XCT revealed greater wet root volume in the P-mix samples and smaller wet root volume with greater fine root formation in the Control samples. High-resolution synchrotron micro-XCT images collected on small root sections confirmed the significant increase in root surface area for Control samples relative to the inoculated ones (Figure 3).

### P Uptake Studied by SR-XRF

**Figure 4:**
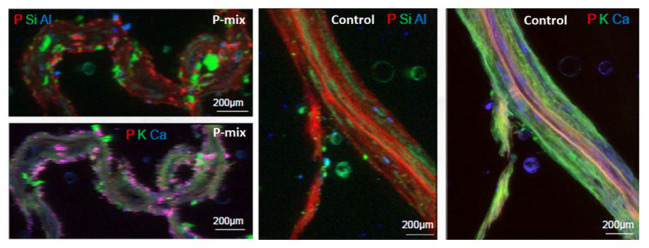
Micro-x-ray fluorescence (μXRF) maps of longitudinal root sections show evidence of P uptake in plant that has the endophytic bacterial strains WP5 and WP42 (“P-mix”). P appears pink in bottom left and right most image due to P and Ca overlapping.

To further confirm and visualize P solubilization by the plant root, we conducted a combination of synchrotron x-ray micro-fluorescence (μXRF) imaging and P K-edge XANES experiments. Roots from poplar samples inoculated with the bacterial consortium and uninoculated samples were harvested for both longitudinal and cross sectioning and used for microprobe (μXRF and μXANES) analyses to investigate P distribution and speciation in the roots. Longitudinal sections showed a marked difference in P distribution for the inoculated plant compared to controls. While the longitudinal sections appeared to show a uniform P distribution across the imaged root section for controls, the plants inoculated with P-mix exhibited distinct hot spots where P accumulated inside the root (Figure 4).

**Figure 5:**
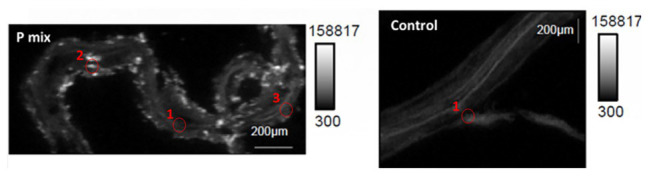
Phosphorus x-ray fluorescence maps (grayscale) in poplar root longitudinal thin sections of the P-mix and Control samples. P in the roots is displayed on the same P intensity scale and same gamma value (white levels, expressed as counts in the maps, are the same). Red circles mark the spots where x-ray absorption near edge structure (XANES) spectra were collected (P-mix Long Spot 1, Spot 2, Spot 3, and Control Long in Supplementary Material).

When the distribution of P in the roots is displayed on the same P intensity scale (Figure 5), we found a significant difference in the P distribution within the root from the endophyte-inoculated plant vs. the control. The P-mix sample also showed a higher P concentration in certain spots when compared to the Control sample. We presume that the Control sample has the homogeneously distributed P from the water-soluble ammonium phosphate in the Hoagland’s solution (Hoagland and Arnon, 1938), while the P-mix samples have the additional, solubilized P from the insoluble phosphate, superimposed on the “background” P present in both groups. See Supplementary Figures S4, S5 for additional μXRF images.

High-resolution x-ray fluorescence microprobe (μXRF) maps collected from inside the roots at the APS were used for a quantitative comparison of P concentrations between P-mix and Control samples. μXRF has been proven to be a useful approach for quantitative analysis of trace elements from environmental and biological samples (Twining et al., 2003; Fittschen and Falkenberg, 2011; Kirker et al., 2017). From quantitative elemental analysis perfomed on 17 different spots for P-mix and Control samples each, we found that the P concentrations inside the roots were consistently greater (by 20–30%) for all P-mix samples than for Control samples (see Supplementary Figure S6 and Supplementary Tables S2–S5).

### Chemical State of P From XANES

**Figure 6:**
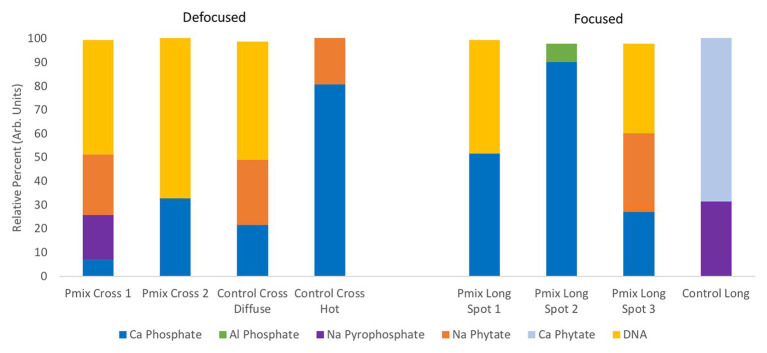
Summary of defocused (bulk characterization) and focused (speciation of the hot spots identified in Figure 5) P K-edge XANES linear combination fit findings collected on longitudinal (i.e., long) and cross sections of P-mix and Control root samples.

To learn about the chemical state of the P in those hot spots in Figure 5, XANES spectra were collected at the P K-edge both in defocused (overall P speciation of the roots) and focused modes (speciation of the hot spots) for root sections. We found species of inorganic and organic phosphates to be present in both the P-mix and Control samples (Figure 6). The inorganic P in both groups is mostly Ca-bound [Ca_3_(PO_4_)_2_, Ca(H_2_PO_4_)_2_, CaHPO_4_, amorphous Ca phosphate, Ca_5_(PO_4_)_3_(OH,F,Cl), and Ca_2_P_2_O_7_]; other inorganic phosphates identified are sodium pyrophosphate (Na_4_P_2_O_7_) and aluminum phosphate (AlPO_4_). The organic P could be identified as being chemically similar to Na‐ or Ca-phytate (C_6_H_18_O_24_P_6_) and DNA (C_x_H_x_O_x_N_x_P).

We found subtle differences between the P-mix and Control samples for P speciation collected in defocused mode (Figure 6; Supplementary Figure S8 and Supplementary Table S6). Overall, P-mix defocused samples were dominated by organic species (DNA and phytate) which comprised 73.5% in Cross 1 and 68% in Cross 2. Control samples were inconsistent, as Control Cross Diffuse organic species represented 77% of total P and Control Cross Hot was only composed of 20.6%. Control Cross Hot was instead dominated by Ca-bound inorganic P (80.8%). Although composed of proportionally smaller amounts, inorganic P in the other defocused samples was also primarily associated with Ca. We examined P hot spots in the P-mix samples using microfocus mode at three locations (see Supplementary Figure S8 and Supplementary Table S6). Our findings show two of the hot spots were dominated by inorganic Ca species with 51.6% (P-mix Long Spot 1) and 90.3% (P-mix Long Spot 2). Taken together, we hypothesize that the homogeneous P background observed in the maps of Figure 5 can be attributed to the readily absorbed ammonium phosphate from the Hoagland’s solution being used by the plant, while the P hot spots seen in the P-mix samples are related to the presence of Ca_3_(PO_4_)_2_ that was taken in with the help of the endophytes. The increased P in the plant is most likely from the increased uptake of solubilized P due to the endophyte activity in solubilizing the Ca_3_(PO_4_)_2_. Why do the microfocus spots look like Ca-bound P? As mentioned above, the once solubilized phosphate may have reacted with the Ca present in the plant becoming insoluble again. We found no evidence that endophytes made the phosphate insoluble. We expect a dynamic relationship of P going into and out of solution within the plant. We note that although the Control samples also had hot spots, they were overwhelmingly a characteristic of the P-mix group.

In summary, these μXRF/XANES results provide additional evidence that the endophytes possessed the capacity to solubilize phosphate. The chemical state of P found inside the root suggests that the solubilized P is a mix of inorganic and organic phosphates. The solubilization of the non-water-soluble phosphate [Ca_3_(PO_4_)_2_] was suggested by the presence of inorganic hot spots with chemistry similar to that of inorganic Ca-phosphates. Since the control plants could not solubilize the Ca_3_(PO_4_)_2_ in the medium, they had less available calcium able to react with the phosphate within root tissue.

### Proteomics Analysis

**Figure 7:**
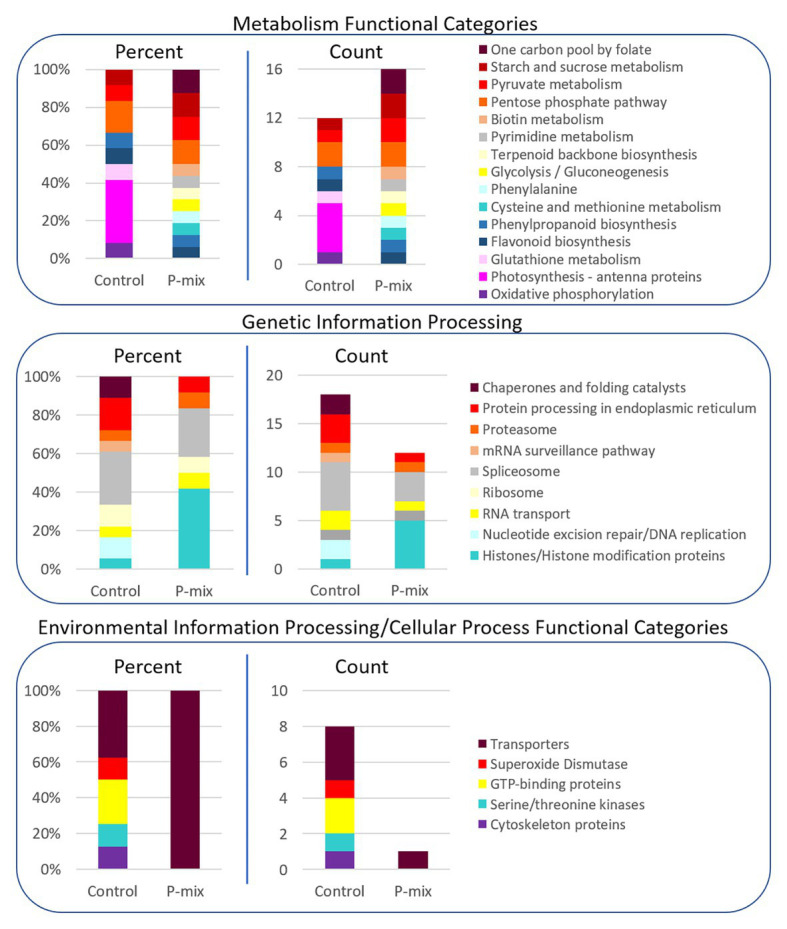
KEGG functional category enrichment distributions displayed as an overall percent or absolute number of proteins identified in each category for those proteins determined to be significantly changed (*p* < 0.05) between Control and P-mix inoculated poplar roots using a *t*-test. A protein was designated to the Control or P-mix group depending on which group it displayed a higher abundance.

In order to further link our phenotypic and image-based P uptake observations to molecular signature of plant-endophyte interactions, and to gain insight into metabolic and system-wide protein changes in inoculated vs. uninoculated poplar roots, we conducted a global proteomics analysis. Phosphate limitation/starvation acts like a stress to plants, stimulating up-regulation of phosphate transporter genes, as well as nutrient sensing signaling networks both locally and systemically (Liu et al., 1998; Chiou and Lin, 2011). As transcriptional regulation only shows potential proteins involved in a process or perturbation, we opted, here, to explore the proteome in order to identify the actual protein machinery present and enriched between P-mix and Control roots. As significant protein enrichment can reveal differences in functional roles between samples, here, we show relative functional category distributions between the P-mix and Control roots. Significant protein enrichment was defined as those proteins increased in abundance in either the P-mix or Control samples and those that passed a Student’s *t*-test with a *p* < 0.05 (Figure 7).

For proteins involved in metabolism (Figure 7, top panel), there were more proteins in total as well as more KEGG functional categories/metabolic pathways enriched in the P-mix inoculated roots than in the Control. This includes higher enrichment of proteins involved in primary carbohydrate metabolism like glycolysis, and starch/sucrose metabolism, which are dependent upon P as cosubstrate. Notable differences between the Control and P-mix group include the large enrichment of oxidative phosphorylation, photosynthesis – antenna proteins, and glutathione metabolism related proteins.

In the Genetic Information Processing analysis (Figure 7, middle panel) we identified the enrichment of several histones in the P-mix inoculated roots. We identified five histones (i.e., POPTR_0011s13490, POPTR_0013s01890, POPTR_0005s04260, POPTR_0018s01310, and Potri_013G028900) significantly increased in abundance in the P-mix inoculated roots and one histone modification protein, POPTR_0019s04940, which was significantly increased in abundance in the Control roots.

We were particularly interested in identifying transporters (related to P transport) enriched in either the P-mix or Control root samples. We observed in the Environmental Information Processing functional category (Figure 7, bottom panel) that the Control samples actually had a greater number of transporters enriched compared to the enrichment of a single transporter in the P-mix samples. The transporters we identified as enriched in the Control samples included an ATPase, an exportin, myosin V, and an aquaporin. However, the only transporter identified as being enriched in the P-mix inoculated samples was a KEGG defined ATPase POPTR_0010s23200, which has some homology to the anion transport protein in *Arabidopsis*. Other ATPases were identified as being significantly changed in the proteomics data but were not functionally annotated by KEGG so were not included in the funcational category comparisons in Figure 7. The ATPase POPTR_0009s12330 was found to be significantly increased in the P-mix trees.

We only identified signaling related proteins to be enriched in the Control samples. However, we identified POPTR_0003s14620 which was significantly increased in abundance in the P-mix roots. This protein has high homology to an *Arabidopsis* tetratricopeptide repeat (TPR)-like superfamily protein. Three kinases, POPTR_0007s14380, POPTR_0001s14410, and POPTR_0013s14080, and one phosphatase, POPTR_0006s09720, were also found significantly enriched in the Control samples.

### Protein Network Analysis

**Figure 8:**
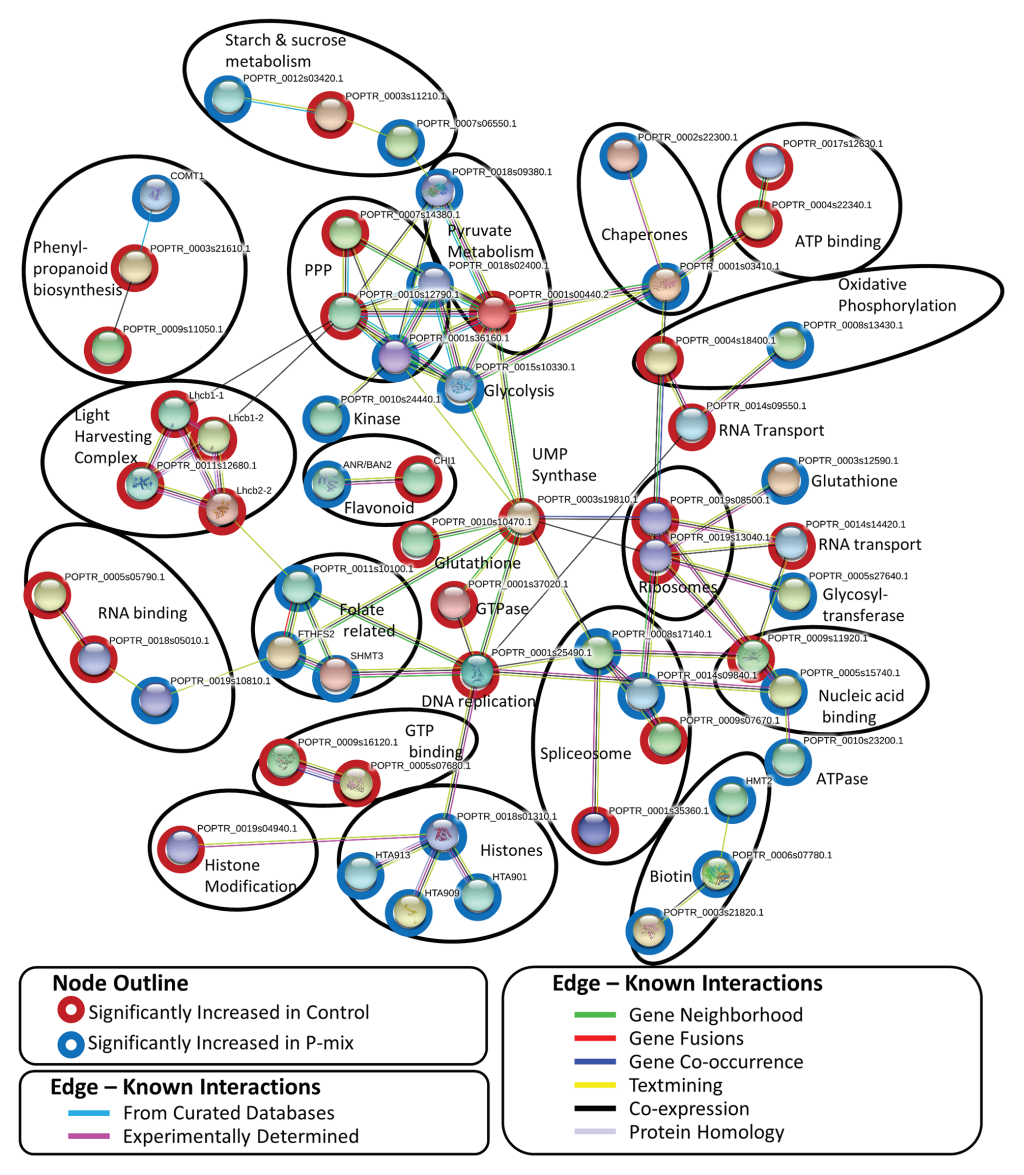
STRING network analysis of proteins found to be significantly changed (*p* < 0.05, *t*-test) between phosphate limited Control and P-mix inoculated poplar roots. Nodes represent proteins and edges represent the protein-protein association. Black ovals designate functional groupings.

A STRING^8^ (Szklarczyk et al., 2019) network analysis was performed on 100 significantly changed proteins found between the Control and P-mix root proteomics analyses. STRING network analysis works to identify known or predicted protein-protein associations between proteins utilizing prior analyses which include known interactions found in curated databases, experimentally determined databases, predicted interactions, including gene neighborhood, gene fusion, and gene co-occurrence analyses, and other predicted interactions based on text-mining, co-expression, and protein homology. Figure 8 shows the interactions between 57 significantly changed poplar proteins found to have at least one protein-protein association in the STRING database. Each node in the figure represent a protein and each edge represents the association. The color of the edge represents the type of association known between the two proteins/nodes. Associations are meant to convey a variety of ways the proteins are related and do not necessarily mean they are physically binding/interacting *in vivo* with one another. Prominent clusters were further grouped using a black circle and the functional category or activity was noted.

## Discussion

Our solubilization experiments confirmed the earlier finding that poplar endophyte strains WP5 and WP42 had the ability to solubilize Ca_3_(PO_4_)_2_ (Khan et al., 2015; Kandel et al., 2017). It is noted that most poplar microbiome studies lack the resolution to determine how common these particular bacterial species are in poplar (Ulrich et al., 2008; Gottel et al., 2011; Shakya et al., 2013; Beckers et al., 2017; Timm et al., 2018; Firrincieli et al., 2020); however, the genera of the two endophyte strains, *Rahnella* and *Burkholderia*, are fairly common culturable isolates from wild *P. trichocarpa* (Doty et al., 2009; Kandel et al., 2017). In a study of the poplar root microbiome, it was found that most of the core rhizosphere OTU’s were within the order of Burkholderiales and Rhizobiales (Shakya et al., 2013). In another study, Burkholderiaceae comprised about 6% of the poplar rhizosphere (Beckers et al., 2017). Members of the *Rahnella* genus were one of the most abundant ASVs in *P. trichocarpa* in a poplar microbiome study that included the environment from which these strains were isolated (Firrincieli et al., 2020). Members of the family of Burkholderiaceae were up to about 20% of the microbial population in that study.

From tomography-based root imaging, we discovered that poplar samples inoculated with the phosphate solubilizing bacteria strains exhibited a root architecture with greater biomass (greater root volume), while the control samples exhibited increased fine root growth. This suggests an increased P uptake in the plants inoculated with the endophytes as plants that have more access to phosphorus typically produce fewer lateral roots (i.e., they produce less overall root surface area; Niu et al., 2013). High-resolution synchrotron micro-XCT images collected on small root sections confirmed the significant increase in root surface area due to fine root formation for control samples relative to the inoculated ones (Figure 3).

The macronutrient P was successfully visualized inside the root by synchrotron μXRF further indicating that the endophytes possessed the capacity to solubilize phosphate, thereby allowing the plant host to gain increased access to this nutrient. The quantitative μXRF based results that the P concentrations were greater in the P-mix samples are consistent with our micro-CT results that suggest markedly different root architecture and root volume for the samples inoculated with the endophytes. More available nutrient was expected to result in greater root mass growth.

X-ray absorption near edge structure studies into the chemical state of the P contained inside the root suggested that phosphorus within the plant is in the form of a mix of inorganic and organic phosphates. The solubilization of non-water-soluble phosphate was suggested by the presence of inorganic hot spots with chemistry similar to that of Ca-phosphates. We found that the inorganic P in both P-mix and Control groups was mostly Ca-bound, while the organic P could be identified as being chemically similar to phytate and DNA. By solubilizing the Ca_3_(PO_4_)_2_ in the media, the endophyte strains make phosphate and calcium, both macronutrients required for growth, more bioavailable to the plant. While the high reactivity of soluble phosphate with calcium is well-known, and the ability of many soil bacteria to solubilize inorganic P *in vitro* has been established, it was unknown why there was little correlation between solubilization ability and impact on plant growth (Collavino et al., 2010; Bashan et al., 2013). Our results point to a possible explanation for this incongruity. Once within the plant, phosphate seems to readily react with the calcium and other metals prevalent in the plant, becoming insoluble again. A benefit of endophytes over rhizobacteria may be that the phosphate could be stored in a non-reactive form inside the plant root and then re-released within the plant by endophytes as needed.

Considering the potential benefits of P solubilization enhanced by endophytes, the ability of WP5 and WP42 endophyte strains to fully solubilize Ca-phosphate could be beneficial to plants in alkaline soils, and the ability to at least partially solubilize Al-phosphate and Fe-phosphate could benefit plants in the more common acidic soils. While Ca-phosphate solubilization is commonly published as a symbiotic trait, Ca_3_(PO_4_)_2_ is actually not difficult for many bacteria to dissolve (Bashan et al., 2013). The mechanism for solubilization of Ca-phosphate is primarily by organic acid production (Chhabra and Dowling, 2017), which is a fairly common trait. In screens of TCP-solubilizing bacteria for plant growth promotion, very few were positive. It is rarer to find Al-phosphate and Fe-phosphate solubilizers (Perez et al., 2007). Since solubility of Fe-phosphate decreases with lower pH, and Al-phosphate has the lowest solubility within pH 5.5 and 4.5, acidification through production of organic acids would not result in P solubilization in these cases (Bashan et al., 2013). The ability of WP5 and WP42 to solubilize, at least to some degree, all three common forms of phosphate is possibly reflective of the environment from which they were isolated. Both strains were isolated from wild poplar growing in primary substrates of cobble and sand with low organic matter. The source of the Snoqualmie River is high alpine snow melt; therefore, the environment is likely to be highly selective for the ability to make all sources of P bioavailable (Doty et al., 2009; Stettler, 2009). The growth data of both strains in ENBRIP do not fully align with this thinking; however, the mechanisms of solubilizing Al and Fe phosphate are less well understood. It may be that cells adhere to these phosphates during the solubilization process. If this is the case, then cells would have settled out with the excess phosphate and would not contribute to the optical density of the media. In support of this hypothesis, we often observed a high degree of cell clumping when dilutions of the phosphate cultures were plated but not from the inoculated no phosphate controls.

We explored the proteome in the plants in order to identify the actual protein machinery present and enriched between P-mix and Control roots. In this study, Control roots could be thought of as P-limited and potentially more stressed compared to the P-mix root where P was more available (by endophytes). An increased chlorosis observed in the Control trees compared to the P-mix trees may be indicative of this increased stess due to P being more limited. However, unfortunately, we did not have enough replicates to monitor these kinds of phenotyping responses.

The higher enrichment of proteins involved in primary carbohydrate metabolism (glycolysis and starch/sucrose metabolism, both dependent upon P as cosubstrate) indicates that the greater uptake of phosphorus brought about by P-solubilizing endophytes stimulated more metabolic pathways than when phosphate was less available and when metabolism presumably was slowed due to the relative limitations of this essential nutrient. The large enrichment of oxidative phosphorylation, photosynthesis – antenna proteins, and glutathione metabolism related proteins in the Control samples is connected to nutrient limitation and abiotic stress in that group. It is documented that sugars are redistributed to the roots when phosphate is less available (Ciereszko et al., 2005; Morcuende et al., 2007; Muller et al., 2007). Greater sugar accumulation would require increased oxidative phosphorylation capability to catabolize the sugars into ATP.

With regards to the histones that were found to be significantly increased in abundance in the P-mix inoculated roots and the one histone modification protein, which was significantly increased in abundance in the Control roots, phosphate availability is known to be involved in chromatin modification (Smith et al., 2010). In *Arabidopsis*, histone H2A.Z was found to regulate phosphate starvation response genes (Smith et al., 2010). Prior studies have shown that reactive oxygen species (ROS) concentrations are increased when P and other nutrients (i.e., nitrogen, potassium, and sulfur) are less available (Shin and Schachtman, 2004; Shin et al., 2005; Schachtman and Shin, 2007; Tyburski et al., 2009). ROS has also been associated with changes in root system architecture (RSA; Shin et al., 2005; Tyburski et al., 2009) and regulation of genes *via* oxidation reduction reactions. As a protection mechanism against ROS, plant cells utilize antioxidant and other oxidation ameliorating molecules, such as glutathione, super oxide dismutase (SOD), and light-harvesting antenna complex proteins (LHCBs), which are also induced in Control plants in this work. While the induction of antenna complex proteins in root tissues (which are devoid of photosynthesis capability) appears unusual, it is documented in the literature that antenna protein expression is coupled to abscisic acid signaling capable of being complexed with carotenoids and xanthophylls as well as chlorophyll, which are very efficient in ROS scavenging (Kobayashi et al., 2013) and have been found to be expressed ubiquitously in all different tissues, including roots of *Arabidopsis* (Smith et al., 2010; Xu et al., 2012). A common feature of endophytes is the ability to scavenge ROS, resulting in lowered stress responses in the host plant (Sessitsch et al., 2012). We previously reported that a consortium of endophyte strains, including strain WP5, reduced ROS levels in poplar (Khan et al., 2016).

Based on our exploration of which transporter proteins showed enrichment in the Control or the P-mix samples, it appears that greater phosphate availability does not necessarily equate into greater diversity of active transporters. Instead it appears as though the Control samples, where P was less available, required the enrichment of a greater number of transporters. One transporter that was identified as being enriched in the P-mix samples, and is related to the anion transport protein in *Arabidopsis*, may serve to aid transport of the phosphate provided by endophytes. The ATPase protein that was found to be significantly increased in the P-mix trees is a four-way junction helicase. Helicases are involved in unwinding DNA during replication. This ATPase might, therefore, play a role in cell replication which could support the increased metabolic activity occurring in the roots of the P-mix trees.

The enrichment of signaling-related proteins in the Control samples indicated that more cell-signaling networks were activated and enriched in the roots when the trees had less P available than when the P-solubilizing endophytes were present. At the same time, the *Arabidopsis* TPR-like superfamily protein, which was significantly increased in abundance in the P-mix roots, is thought to be involved in the regulation of different cellular functions and plant hormone signaling like TPR proteins (Schapire et al., 2006). Specifically, these proteins have been found to be essential for abscisic acid, ethylene, cytokinin, gibberellin, and auxin which are all hormones involved in P sensing responses (Chiou and Lin, 2011).

In protein network analyses, centrality is a proxy for essentiality or importance. The protein/node uridine monophosphate (UMP) synthase (UMPS; POPTR_0003s19810) displayed the highest betweenness centrality. UMPS catalyzes the formation of uridine monophosphate which is a building block of RNA and pyrimidine synthesis. UMPS may also play a role as a negative regulator in increased availability of sugar in the roots. It is well-documented that when plants experience less phosphate in the roots, starch in shoot tissue is broken down and the released sugars are mobilized to the roots (Ciereszko et al., 2005; Morcuende et al., 2007; Muller et al., 2007). When UMPS expression was decreased in potato tubers, there were increased conversions of sucrose to starch and cell wall synthesis (Geigenberger et al., 2005). In our network analysis, UMPS displayed the highest betweenness centrality connecting major protein clusters related to genetic information processing (e.g., nucleic acid replication, chromatin remodeling, spliceosome, and ribosomal proteins) and carbohydrate metabolism. We hypothesize that disruption of this protein may cause interruption in sugar metabolism related to P availability.

In summary, the proteomics data revealed that more proteins involved in a diverse array of metabolic activities were enriched in the P-mix roots, whereas proteins related to abiotic stress, cell-signaling, and ROS amelioration were enriched in the Control samples which experienced less P availability.

Finally, we note that in order to visualize the effects of the inoculated strains on poplar, it was essential to begin with internally sterile plants. It is unknown how other members of the plant microbiome may affect the phosphorous solubilization performed by the two strains, WP5 and WP42. Microbial community dynamics can be complex, with both positive and negative interactions at play. However, these microbial interplays on which strains solubilize the phosphates would likely not affect the impact of bacterially solubilized phosphate on the host plant. Since bioavailable phosphate is an essential nutrient and solubilization is an exported activity, it is a “public good” subject to microbial “cheating” (recently reviewed in Smith and Schuster, 2019).

Further research is required to determine the mechanisms by which WP5 and WP42 solubilize the three phosphates. Also, in order to screen specific inorganic-organic associations related to P nutrient solubilization processes and to learn about the elemental distribution and speciation of the elements P and O on the nanoscale, future studies will employ scanning transmission x-ray microscopy in the soft x-ray energy region at 25–50 nm resolution combined with XANES.

## Conclusion

Using laboratory and synchrotron XCT, synchrotron x-ray fluorescence spectromicroscopy and proteomics we found direct evidence of endophyte-promoted phosphorus uptake in poplar. Root imaging by XCT revealed greater root volume in the samples inoculated with the endophytes and smaller wet root volume with greater fine root formation in the control samples. This suggested an increased P uptake in the inoculated plants as plants that have more access to phosphorus typically produce fewer lateral roots. Using synchrotron x-ray fluorescence spectromicroscopy, we visualized the nutrient phosphorus inside poplar roots inoculated by the selected endophytes and found the phosphorus in both forms of organic and inorganic phosphates inside the root. Proteomics characterization on poplar roots coupled with protein network analysis revealed novel proteins and metabolic pathways with possible involvement in endophyte enriched phosphorus uptake, cell signaling, and metabolism. Our results have a significant implication: phosphate taken up by plants can form insoluble phosphate compounds within the plant; and therefore, rhizobacteria with the ability to solubilize P exclusively outside the plant tissue in soil may be at a disadvantage compared with endophytes. Being within the plant, endophytes may have the ability to continue re-releasing the phosphate for continued plant growth. In addition, since the endophytic bacteria in this study possess at least some ability to promote solubilization of all three common forms of phosphate, i.e., Ca-, Al, and Fe-phosphate, they may have an advantage as bioinoculants in both alkaline and acidic soils. In all, these findings suggest an important role of endophytes for phosphorus acquisition and provide a deeper understanding of the symbiotic associations between poplar and the endophytic bacteria.

## Data Availability Statement

The datasets presented in this study can be found in online repositories. The names of the repository/repositories and accession number(s) can be found at: http://proteomecentral.proteomexchange.org/cgi/GetDataset?ID=PXD017325.

## Author Contributions

TV developed the study, carried out the x-ray-based (tomography and spectroscopy) experimental work, and prepared the first draft of the manuscript. KKH performed the proteomics characterization work with help from CDN, analyzed all proteomics data, and provided critical inputs for the study as well as to the writing of the manuscript. AHA directed the root collection process performed by TEW and provided critical inputs for the study as well as during preparation of the manuscript. AWS performed all of the microbial experiments and subsequent data analysis, prepared Figures 1 and 2 and assisted in manuscript preparation. MEB performed critical analysis of the XANES data and provided critical inputs to its discussion in the manuscript. RKC prepared root thin sections for x-ray spectroscopy and prepared the soil for ICP-MS characterization. LRR and AKB helped with the tomography data processing and analysis. SCF helped to collect the micro-XRF and XANES data, created some of the micro-XRF maps (from the ALS), contributed to the XANES analysis as well as to the writing of the manuscript. OA helped to collect some of the micro-XRF maps (from the APS) and helped with related data analysis. DYP helped to collect the synchrotron XCT data. JRH performed the preliminary microbial experiments. SLD provided the plant samples with and without the microbial strains, assisted in the microbial experiments, provided critical inputs for the study as well as during preparation of the manuscript.

### Conflict of Interest

The authors declare that the research was conducted in the absence of any commercial or financial relationships that could be construed as a potential conflict of interest.

## Footnotes

^1^https://genome.jgi.doe.gov/

^2^https://www.kegg.jp/

^3^http://geneontology.org/

^4^https://genome.jgi.doe.gov/

^5^https://omics.pnl.gov/software/infernordn

^6^http://mev.tm4.org/

^7^https://string-db.org

^8^https://string-db.org/

## Supplementary Material

All proteomics data can be accessed through MassIVE (accession # MSV000084860) and ProteomeXchange (accession # PXD017325).
