# Supplementary material for: Endophyte-Promoted Phosphorus Solubilization in Populus
Source: Front Plant Sci. 2020 Oct 21;11:567918. doi: 10.3389/fpls.2020.567918 (PMC7609660; doi:10.3389/fpls.2020.567918)
Supplement: Supplementary file 1 [file Data_Sheet_1.pdf]

Supplementary Material for:

Endophyte-Promoted Phosphorous Solubilization in *Populus*

Tamas Varga[1*], Kim K. Hixson[1], Amir H. Ahkami[1], Andrew W. Sher[2], Morgan E. Barnes[3], Rosalie K. Chu[1], Anil Krishna Battu[1], Carrie D. Nicora[4], Tanya E. Winkler[4], Loren R. Reno[1], Sirine C. Fakra[5], Olga Antipova[6], Dilworth Y. Parkinson[5], Jackson R. Hall[2], and Sharon L. Doty[2]

[1]Environmental Molecular Sciences Laboratory, Earth and Biological Sciences Directorate, Pacific Northwest National Laboratory, Richland, WA, 99352

[2]School of Environmental and Forest Sciences, College of the Environment, University of Washington, Seattle, WA, 98195

[3]Environmental Systems Graduate Group, University of California, Merced, CA, 95343

[4]Earth and Biological Sciences Directorate, Pacific Northwest National Laboratory, Richland, WA, 99352

[5]Advanced Light Source, Lawrence Berkeley National Laboratory, Berkeley, CA, 94720

[6]Advanced Photon Source, Argonne National Laboratory, Lemont, IL, 60439

*Email: tamas.varga@pnnl.gov

Table of Contents: Page

Soil P content analysis by ICP-MS (Figure S1) 2
Pictures of P-mix and Control plant samples (Figure S2) 3
Root length, root weight, and leaf weight data (Table S1) 4
Poplar root samples mounted on a sample holder for the microprobe at ALS 5
Micro-XRF images (Figures S4 and S5) 6
P quantitation from micro-XRF maps (Figure S6) 8
Tables for comparing P concentrations in P-mix and Control samples (Tables S2-S5) 9
P K-edge XANES spectra of inorganic and organic reference compounds (Figure S7) 11
Linear combination fits of P-mix and Control samples (Figure S8) 12
Summary of all Linear Combination Fitting results (Table S6) 14
Proteomics data repository info 15

SampleName	LabNumber	Analyte	FinalResult	Units	EQL
Soil-1-TV	1907002-01	Aluminum	28400	ug/L	.
Soil-2-TV	1907002-02	Aluminum	44100	ug/L	49.4
Soil-1-TV	1907002-01	Barium	158	ug/L	9.85
Soil-2-TV	1907002-02	Barium	219	ug/L	9.85
Soil-1-TV	1907002-01	Boron	281	ug/L	75.6
Soil-2-TV	1907002-02	Boron	204	ug/L	75.6
Soil-1-TV	1907002-01	Calcium	20200	ug/L	101
Soil-2-TV	1907002-02	Calcium	12400	ug/L	101
Soil-1-TV	1907002-01	Chromium	42	ug/L	6.95
Soil-2-TV	1907002-02	Chromium	691	ug/L	6.95
Soil-1-TV	1907002-01	Copper	46.4	ug/L	23.5
Soil-2-TV	1907002-02	Copper	89.3	ug/L	23.5
Soil-1-TV	1907002-01	Iron	14000	ug/L	30
Soil-2-TV	1907002-02	Iron	41600	ug/L	30
Soil-1-TV	1907002-01	Magnesium	6070	ug/L	8.11
Soil-2-TV	1907002-02	Magnesium	4360	ug/L	8.11
Soil-1-TV	1907002-01	Manganese	769	ug/L	7.18
Soil-2-TV	1907002-02	Manganese	3490	ug/L	7.18
Soil-1-TV	1907002-01	Phosphorus	1690	ug/L	122
Soil-2-TV	1907002-02	Phosphorus	2680	ug/L	122
Soil-1-TV	1907002-01	Potassium	52000	ug/L	483
Soil-2-TV	1907002-02	Potassium	49000	ug/L	483
Soil-1-TV	1907002-01	Silicon	136000	ug/L	164
Soil-2-TV	1907002-02	Silicon	180000	ug/L	164
Soil-1-TV	1907002-01	Sodium	8370	ug/L	134
Soil-2-TV	1907002-02	Sodium	7140	ug/L	134
Soil-1-TV	1907002-01	Strontium	116	ug/L	18.8
Soil-2-TV	1907002-02	Strontium	115	ug/L	18.8
Soil-1-TV	1907002-01	Sulfur	2580	ug/L	143
Soil-2-TV	1907002-02	Sulfur	1950	ug/L	143
Soil-1-TV	1907002-01	Titanium	487	ug/L	7.1
Soil-2-TV	1907002-02	Titanium	737	ug/L	7.1
Soil-1-TV	1907002-01	Vanadium	28.9	ug/L	25.6
Soil-2-TV	1907002-02	Vanadium	81.9	ug/L	25.6
Soil-1-TV	1907002-01	Zinc	59.5	ug/L	18.5
Soil-2-TV	1907002-02	Zinc	68.9	ug/L	18.5
Soil-1-TV	1907002-01	Zirconium	44	ug/L	5.72
Soil-2-TV	1907002-02	Zirconium	57.5	ug/L	5.72

Figure S1: ICP-MS analysis of the total P concentration for the soil that contained a P-mix sample (Soil-1-TV) was 1690 µg/L, while for the soil that contained a control sample (Soil-2-TV) it was 2680 µg/L.

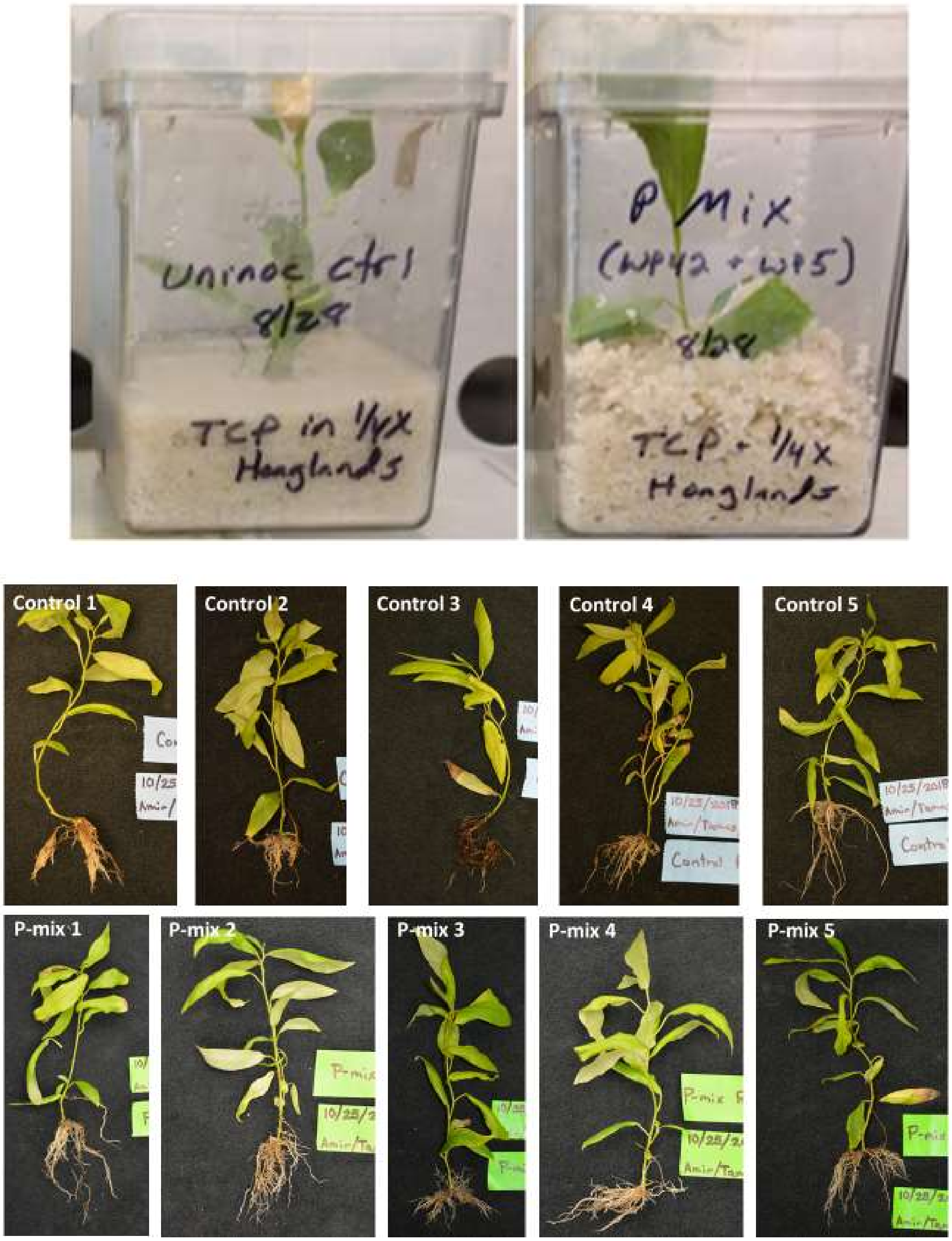

Figure S2: Photos taken of two representative (Control and P-mix) samples as grown in the soil and each poplar sample harvested for root mass, morphology, and proteomics measurements.

Table S1: Longest root length, root mass, and leaf mass values for all P-mix and Control samples.

Sample	Longest root length (cm)	Root weight (g)	Leaf weight (g)
P-mix 1	8.8	0.36	0.89
P-mix 2	5.1	0.36	0.68
P-mix 3	3.9	0.31	1.02
P-mix 4	5.8	0.27	0.84
P-mix 5	6.3	0.23	0.89
Control 1	6.5	0.32	0.64
Control 2	4.5	0.30	0.73
Control 3	8.0	0.56	0.83
Control 4	5.0	0.17	0.82
Control 5	7.8	0.42	1.02
Max:	8.8	0.36	1.02
Average:	6.0	0.31	0.86
Total:	29.9	1.53	4.32
Max:	8.0	0.56	1.02
Average:	6.4	0.35	0.81
Total:	31.8	1.77	4.04

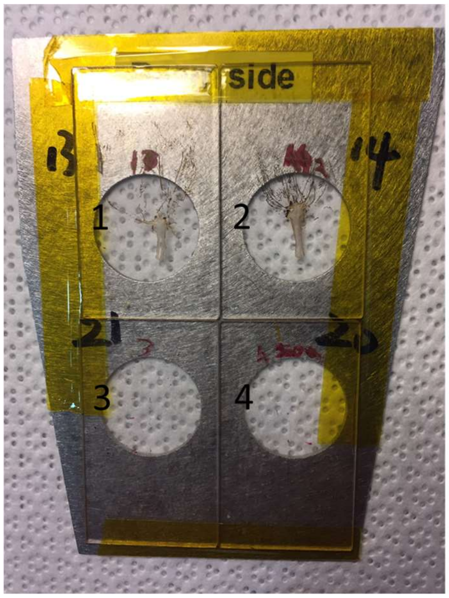

- 1: Poplar control longitudinal
- 2: Poplar P-mix longitudinal
- 3: Poplar P-mix cross
- 4: Poplar control cross

Figure S3: Thin sections of Poplar root samples mounted on a sample holder for the microprobe at ALS.

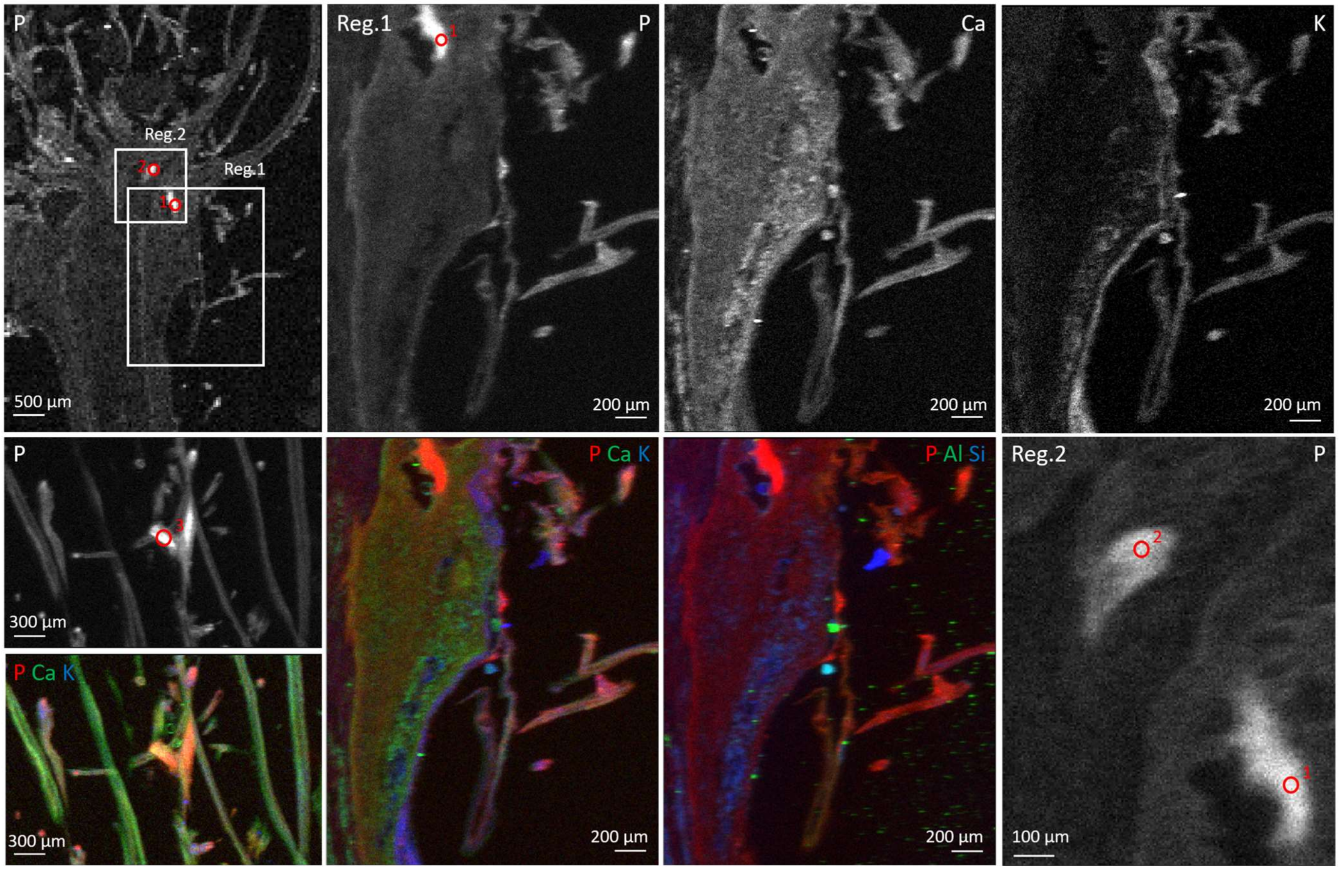

Figure S4: Grayscale images and tricolor maps from fine fluorescence mapping of thin sections of sample "Poplar P-mix longitudinal". The tricolor maps show elements P, Al, Si, Ca, and K. Spots labeled 1, 2, and 3 mark different P hot spots.

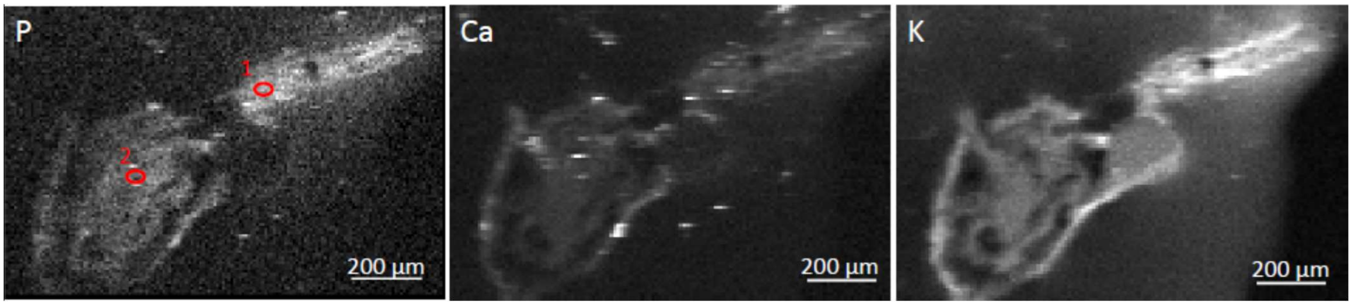

Figure S5: Grayscale images from fine (microfocus) fluorescence mapping of thin sections of sample “Poplar P-mix cross” (cross sectional slice). The maps show elements P, Ca, and K. Spots labeled 1 and 2 mark spots where the “Pmix Cross” spectra were taken.

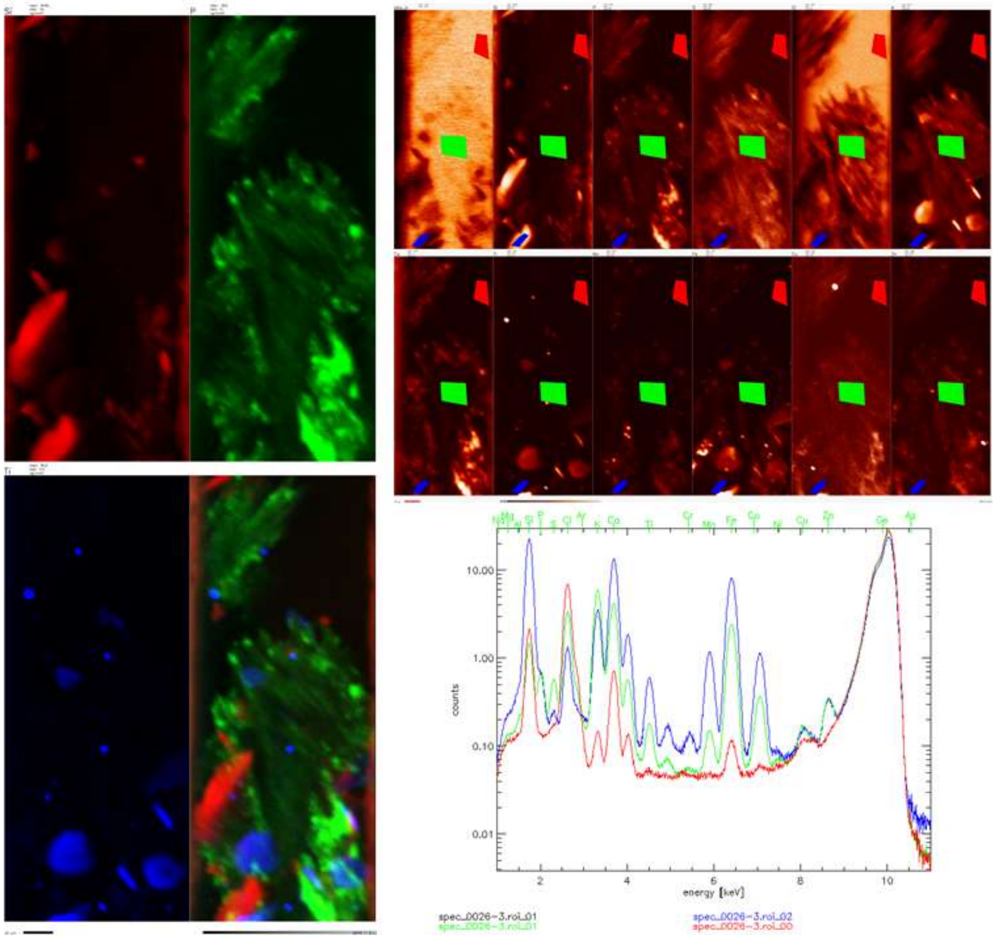

Figure S6: P concentrations inside the roots determined from all high-resolution micro-XRF maps collected at beamline 2-ID-E at the APS using the MAPS software. P indicated by green on raw maps (left) and green line on graph. P concentrations were determined from 2 Regions of Interest (ROIs), from inside the root (green squares) and substrate background from outside the root (red squares). P concentration was found to be consistently greater (by 20-30 %) for all P-mix samples than for controls.

Table S2: Micro-XRF based quantitation of P in a longitudinal root section from P-mix sample 1 (compared with Control).

Sample1-longitudinal Pmix, data summary

Poplar P-mix longitud (sample 1)							
Scan ID	Scan window (um)	Region area (um^2)	P conc. (corr. ug/cm^2)	Ca conc. (corr.)	K conc. (corr.)	Fe conc. (corr.)	Zn conc. (corr.)
076	0.2 x 0.3	335.1	61.33	167.13	16.38	10.91	0.09
077	0.2 x 0.3	638.4	102.75	227.27	16.16	34.02	0.87
078	2 x 5 ?	257.8	71.84	197.77	63.06	43.12	
079	0.2 x 0.3	535.7	58.54	140.13	46.4	23.73	
080	0.2 x 0.3	538.6	14.32	18.47	53.35	7.42	
0.81	0.2 x 0.3	243.5	24.08	22.07	29.7	3	
082	0.2 x 0.3	676.9	44.94	45.8	100.56	4.28	
		Average concentrations:	53.97	116.95	46.52	18.07	0.48
		Averages from control:	45.48	51.48	174.70	2.68	

Table S3: Micro-XRF based quantitation of P in a longitudinal root section from P-mix sample 2 (compared with Control).

Sample2- cross section P-mix, data summary

Poplar P-mix Cross (sample 2)							
Scan ID	Scan window (um)	Region area (um^2)	P conc. (corr. ug/cm^2)	Ca conc. (corr.)	K conc. (corr.)	Fe conc. (corr.)	Zn conc. (corr.)
022	0.2 x 0.3	147.3	32.07	362.01	54.59	18.73	0.23
023	0.2 x 0.3	264.6	93.08	132.76	210.85	5.83	0.4
024	0.2 x 0.3	534.8	182.56	385.59	138.03	17.78	0.7
025	0.2 x 0.3	779.8	138.75	270.65	90.86	13.79	0.45
026	0.2 x 0.3	819.3	43.07	35.31	76.13	6.52	0.35
027	0.2 x 0.3	329.46	123.74	296.52	89.74	18.3	0.53
028	0.2 x 0.3	461.5	212.57	450.74	88.9	22.7	1.2
029	0.2 x 0.3	253.7	84.27	89.01	113.14	14.17	0.68
029-b	0.2 x 0.3	125.6	82.48	69.85	124.7	13.91	0.82
030	0.2 x 0.3	786.5	136.65	312.09	64.12	16.6	0.5
		Average concentrations:	112.92	240.45	105.11	14.83	0.59
		Averages from control:	88.81	157.01	254.84	5.78	

Table S4: Micro-XRF based quantitation of P in a longitudinal root section from Control sample 3.

Sample3-longitudinal Control, data summary

Poplar Control longitud (sample 3)						
Scan ID	Scan window (um)	Region area (um^2	P conc. (corr. ug/cm^2	Ca conc. (corr.)	K conc. (corr.)	Fe conc. (corr.)
091	0.2 x 0.3	111.2	44.06	73.96	208.36	1.79
092	0.2 x 0.3	1461.1	19.57	39.85	127.21	2.49
093	0.2 x 0.3	517.6	50.27	35.05	101.11	3.15
094	0.2 x 0.3	332.8	26.6	52.97	119.57	3.22
095	0.2 x 0.3	347.3	68.54	34.31	182.56	1.59
096	0.2 x 0.3	192.9	63.81	72.74	309.38	3.82
		Average concentrations:	45.48	51.48	174.70	2.68

Table S5: Micro-XRF based quantitation of P in a longitudinal root section from Control sample 4.

Sample4- cross section Control, data summary

Poplar Control Cross (sample 4)						
Scan ID	Scan res. (um)	Region area (um^2)	P conc. (corr. ug/cm^2)	Ca conc. (corr.)	K conc. (corr.)	Fe conc. (corr.)
053	0.2 x 0.3	149.4	52.41	53.46	271.11	6.85
054	0.2 x 0.3	114.7	46.52	71.89	182.93	3.54
055	0.2 x 0.3	409	168.38	297.13	194.36	16.58
057	0.2 x 0.3	141.48	51.63		186.82	1.48
058	0.2 x 0.3	32.5	79.69	127.02	222.09	7.79
059	0.2 x 0.3	76.3	146.93	322.92	250.06	8.36
060	0.2 x 0.3	54.5	150.21	281.19	311.6	5.67
062	0.2 x 0.3	155.9	58.03	86.2	295.93	4.19
063	0.2 x 0.3	114.3	48.58	80.57	186.43	4
064	0.2 x 0.3	47.9	81.7	164.93	139.13	6.15
065	0.2 x 0.3	133.9	81.2		365.58	1.09
066	0.2 x 0.3	169.7	100.49	84.82	452.04	3.7
		Average concentrations:	88.81	157.01	254.84	5.78

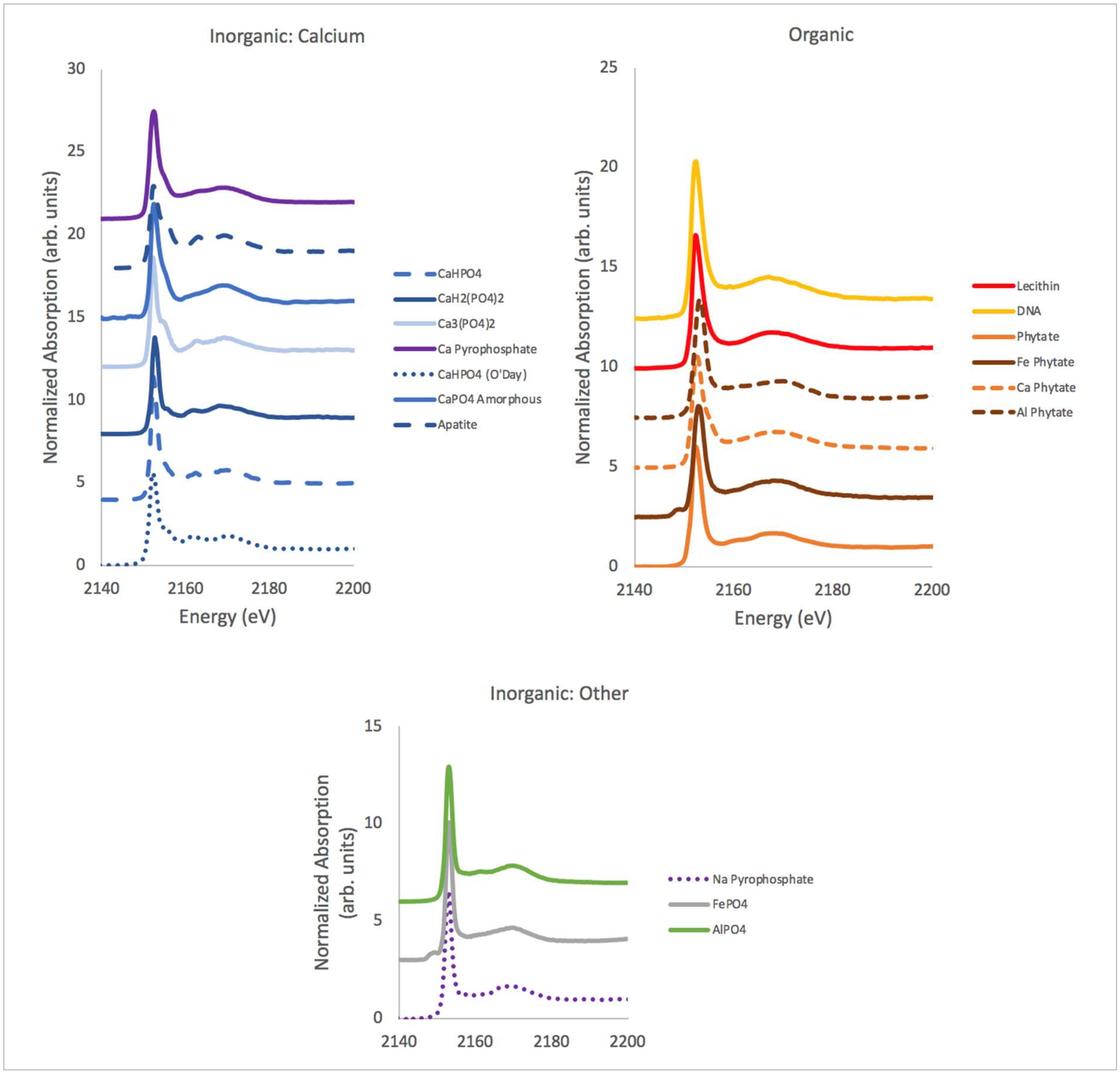

Figure S7: P K-edge XANES spectra of inorganic and organic reference compounds collected at the ALS or acquired through other studies (Barnes M, O'day P, Young R, Hart SC, Berhe AA. 2019. Aeolian particulate matter as a source of bioavailable phosphorus to Sierra Nevada soil. *Unpublished*, and O'day P, Nwosu UG, Barnes M*, et al.*, 2020. Phosphorus Speciation in Atmospherically Deposited Particulate Matter and Implications for Terrestrial Ecosystem Productivity. *Environmental Science & Technology*, accepted). Calcium phosphate ($CaHPO_4$) was used to calibrate sample spectra to the reference compound library of the other studies.

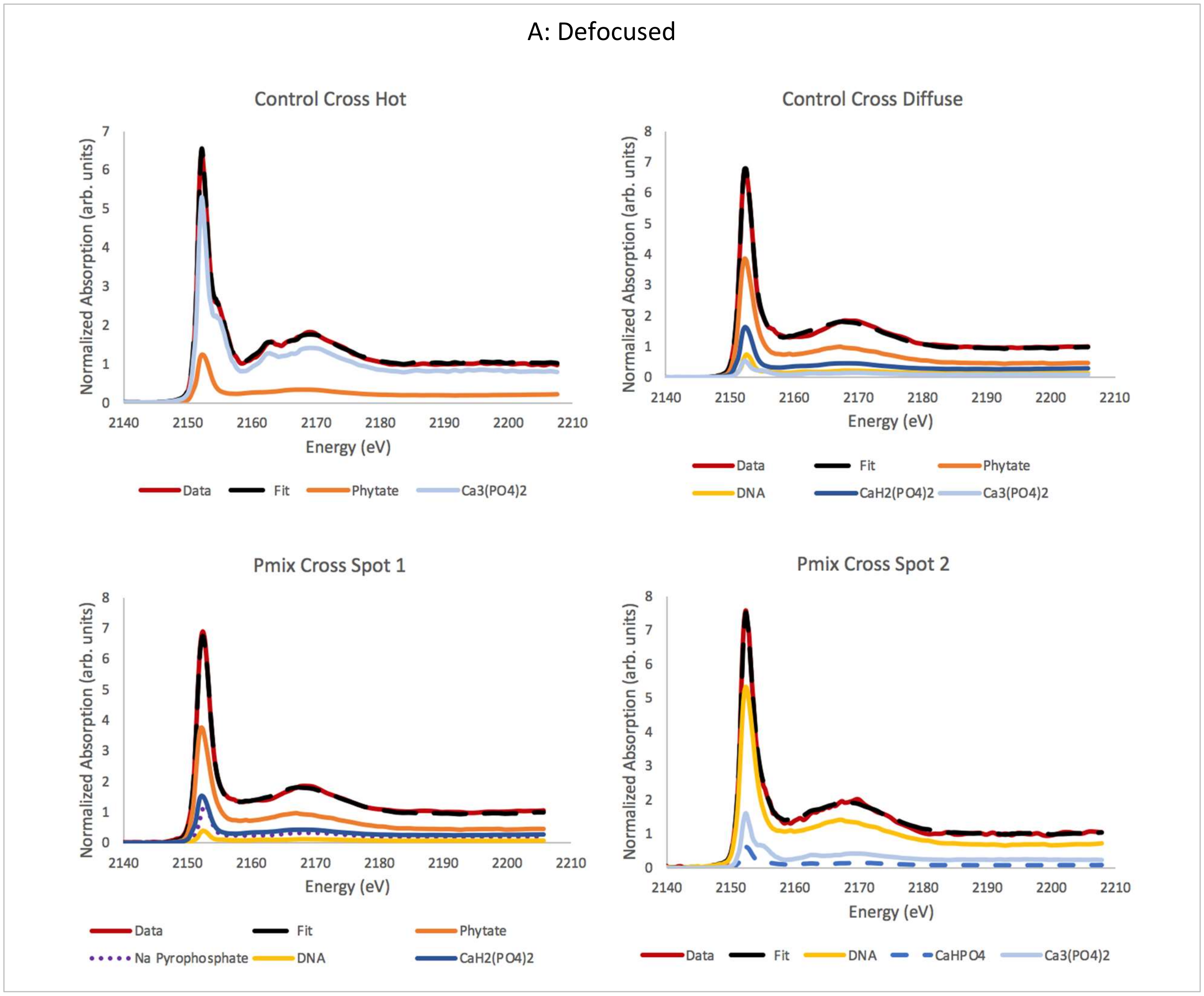
A: Defocused
Control Cross Hot
Normalized Absorption (arb. units)
Energy (eV)
Data
Fit
Phytate
Ca3(PO4)2
Control Cross Diffuse
Normalized Absorption (arb. units)
Energy (eV)
Data
Fit
Phytate
DNA
CaH2(PO4)2
Ca3(PO4)2
Pmix Cross Spot 1
Normalized Absorption (arb. units)
Energy (eV)
Data
Fit
Phytate
Na Pyrophosphate
DNA
CaH2(PO4)2
Pmix Cross Spot 2
Normalized Absorption (arb. units)
Energy (eV)
Data
Fit
DNA
CaHPO4
Ca3(PO4)2

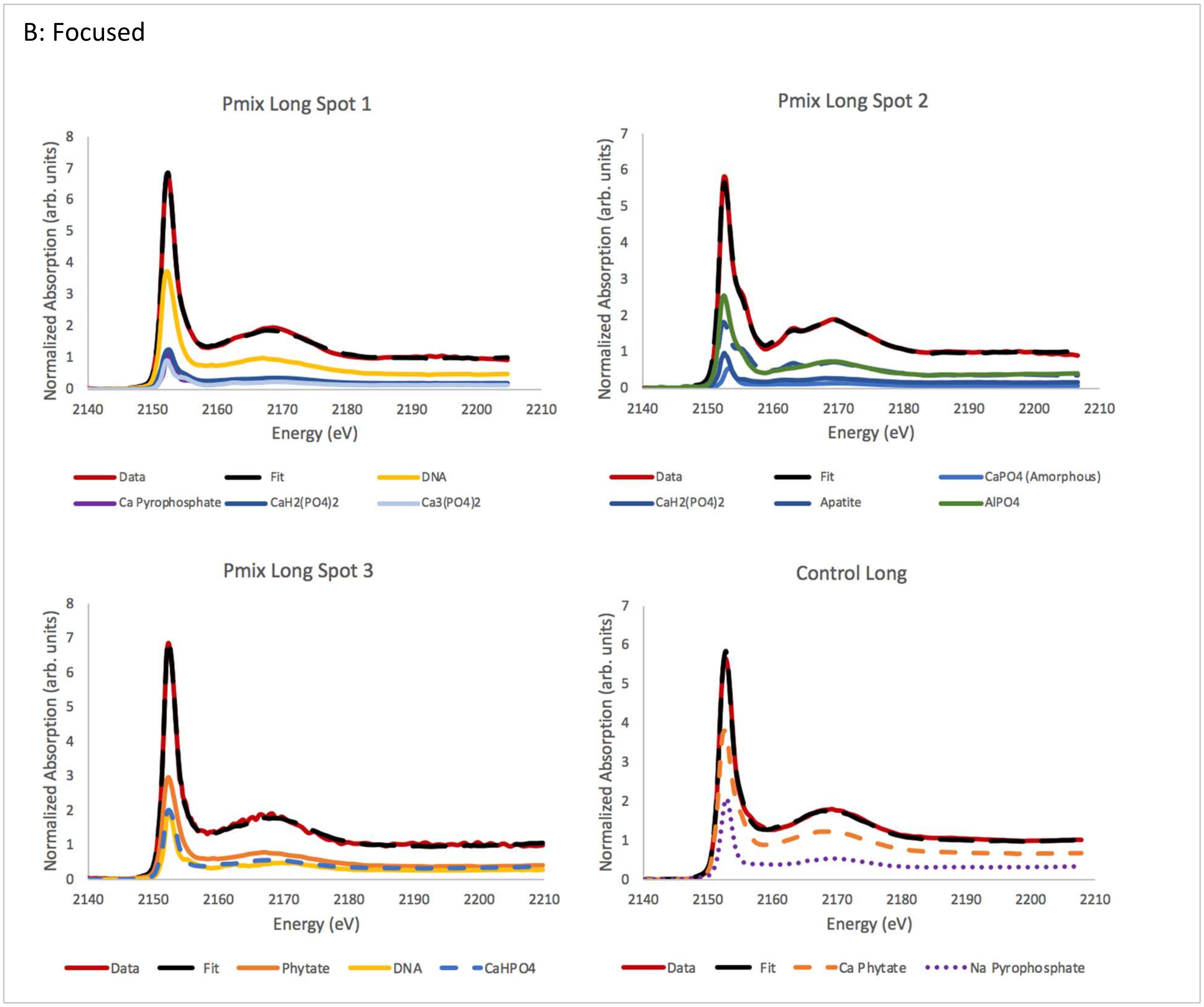

Figure S8 Linear combination fits of P-mix and Control samples collected for P K-edge XANES in defocused (A.) and focused (B.) modes to determine P speciation on thin sections of Poplar roots (Long = longitudinal section; Cross = cross section) using the full reference compound library. Samples were calibrated, normalized, and background subtracted prior to fitting.

Table S6: Summary of focused and defocused P K-edge XANES linear combination fits for Pmix and Control root sections (Long= longitudinal section; Cross= cross section). R-factor: statistical goodness-of-fit equal to ($\sum$ (data - fit)^2 / $\sum$ (data)^2).

Sample	Collection Mode	$Ca3(PO_4)_2$	$CaHPO_4$	$CaH_2(PO4)_2$	Apatite	$CaPO_4$ Amorphous	$AlPO_4$	Ca Pyro	Na Pyro	Phytate	Ca Phytate	DNA	Total	R-factor
P-mix Cross Spot 1	Defocused			7.0					18.8	25.5		48.0	99.3	0.0012629
P-mix Cross Spot 2	Defocused	24.3	8.5									68.0	100.8	0.0017210
P-mix Long Spot 1	Focused	13.5		18.7				19.4				47.9	99.5	0.0005615
Pmix Long Spot 2	Focused			16.6	37.0	36.7	7.5						97.8	0.0042263
P-mix Long Spot 3	Focused		27.1							33.0		37.8	97.9	0.0017315
Control Cross Diffuse	Defocused	8.5		13.2						27.3		49.7	98.8	0.0016437
Control Cross Hot	Defocused	80.8								20.6			101.5	0.0011329
Control Long	Focused								31.4		68.8		100.3	0.0020432

Proteomics data

All relevant raw files, worked up files and TMT channel designations have been uploaded to MassIVE (https://massive.ucsd.edu/) and ProteoExchange (http://www.proteomexchange.org/). The data has been submitted and assigned a MassIVE accession: MSV000084860 and for the ProteoExchange site the accession is PXD017325.
